# Insights into Chemical Diversity and Potential Health-Promoting Effects of Ferns

**DOI:** 10.3390/plants13182668

**Published:** 2024-09-23

**Authors:** Ashaimaa Y. Moussa, Jinhai Luo, Baojun Xu

**Affiliations:** 1 Department of Pharmacognosy, Faculty of Pharmacy, Ain Shams University, Abbassia, Cairo 11566, Egypt; ashaimaa_yehia@pharma.asu.edu.eg; 2 Food Science and Technology Program, Department of Life Sciences, BNU-HKBU United International College, 2000 Jintong Road, Tangjiawan, Zhuhai 519087, China; luojinhai@uic.edu.cn

**Keywords:** pteridophytes, phytochemicals, nutrients, bioactivity, health benefits

## Abstract

The scientific community is focusing on how to enhance human health and immunity through functional foods, and dietary supplements are proven to have a positive as well as a protective effect against infectious and chronic diseases. Ferns act as a taxonomical linkage between higher and lower plants and are endowed with a wide chemical diversity not subjected to sufficient scrutinization before. Even though a wealth of traditional medicinal fern uses were recorded in Chinese medicine, robust phytochemical and biological investigations of these plants are lacking. Herein, an extensive search was conducted using the keywords ferns and compounds, ferns and NMR, ferns and toxicity, and the terms ferns and chemistry, lignans, *Polypodiaceae*, NMR, isolation, bioactive compounds, terpenes, phenolics, phloroglucinols, monoterpenes, alkaloids, phenolics, and fatty acids were utilized with the Boolean operators AND, OR, and NOT. Databases such as PubMed, Web of Science, Science Direct, Scopus, Google Scholar, and Reaxys were utilized to reveal a wealth of information regarding fern chemistry and their health-promoting effects. Terpenes followed by phenolics represented the largest number of isolated active compounds. Regarding the neuroprotective effects, *Psilotium*, Polypodium, and Dryopteris species possessed as their major phenolics component unique chemical moieties including catechins, procyanidins, and bioflavonoids. In this updated chemical review, the pharmacological and chemical aspects of ferns are compiled manifesting their chemical diversity in the last seven years (2017–2024) together with a special focus on their nutritive and potential health-promoting effects.

## 1. Introduction

The history of vascular cryptogams, known as pteridophytes, comprises a wealth of traditional uses and applications practiced by native ethnic people all around the world. Among cryptogams or free-sporing plants, including around 12,000 species in tropical and subtropical regions, ferns rather than lycophytes are more closely related to gymnosperms. Among vascular plants, ferns are the second species-rich clade after angiosperms. Ferns have exhibited many biological effects such as antihyperglycemic [1,2], trypanocidal [3], hepatoprotective, and anti-inflammatory activities. Phytochemical components reported in ferns encompass fatty acids, carotenoids, terpenoids, and polyphenols and are particularly implicated in current age-related diseases due to their marked antioxidant and free radical scavenging power. This was noted in populations largely consuming polyphenols in their diet [4].

Fern’s distribution differs from other plants, as angiosperms are restricted and lower in number, with research groups having limited access; thus, reported information about them is far from enough. A common feature noticed in the production of natural products from ferns was their medicinal uses and ethnobotanical benefits to people where they were located; therefore, their ecological adaptation and chemical diversity are noteworthy. Based on their botanical classification, pteridophytes were unique phylogenetically in their biosynthetic genes from other plants, which made their compounds distinct from other natural sources both structurally and functionally [5].

One example of an imminent world problem is antibiotic resistance, which is more common than ever before and is on the rise, with more than 2.8 million cases in the US resulting in about 35 thousand deaths, per the Centers for Disease Control and Prevention (CDC) annual report of 2019 [6,7]. Ferns were historically established as antibiotic agents dating back to 1980 when the water and alcoholic extracts of 114 pteridophytes were screened for their antimicrobial effects, and about 64% of the samples showed bactericidal activity against Gram positive, Gram negative, and fungal strains; to mention just a few, *Dryopteris cochleate* (D.Don) C.Chr.; *Leptochillus decurrens Blume*.; *Phymatodes ebenipes* (Hook.), *Polypodium irioides* Poir.; *Pyrrosia mannii* (Giesenh.) Ching.; and *Microsorium alternifolium* (Willd.) Copel. had activity [8]. Additionally, ferns have inherited the unique ability to adapt to extreme environments, from the Rocky Mountains and deserts to misty tropical and subtropical regions, owing to their protected spores and modified leaf shapes, which have honed their chemical diversity and provided us with an arsenal of promising bioactive compounds. Thus, fern research could contribute effectively to solving, among other problems, the antibiotic resistance dilemma. This has motivated researchers to embark on more and more studies to unveil the unexplored nature of ferns as a promising solution for the world’s problems [9]. Ferns are evolutionary forerunners compared to gymnosperms and angiosperms, with a genetic machinery that favors chemical diversity and unusual skeletons [9].

In this study, we introduce a chemical review highlighting the most recent isolated phytochemical compounds from ferns between 2017 and 2023 with a critical discussion of their novel aspects and biological activities. Previous reports on ferns were few, in which some discussed taxonomy [10] or the culinary uses of edible ferns in China [11]; moreover, Soeder studied the phytochemical components of known ferns [12]. Cao et al. prepared a compilation of ferns’ compounds isolated from 1980 to 2017 [5], yet incorrectly included lycophytes as Selaginella species under the fern classification. However, scientific interest in fern research has risen dramatically, and more than 300 compounds are reviewed here, most of which are unprecedented.

The main aim of this study is to review the isolation and characterization of more than 300 bioactive compounds from ferns from seven different chemical classes and their reported medicinal effects in the previous six years, thus pointing out the significance of fern research and paving the way for more funding for drug discovery from ferns. Furthermore, the health and nutritive benefits presented by ferns as well as their toxicity concerns are summarized.

## 2. Methodology

### Paper Selection Criteria

In this survey, an extensive search was conducted using the keywords ferns and compounds, ferns and NMR, and ferns and toxicity, and the terms ferns and chemistry, lignans, *Polypodiaceae*, NMR, isolation, bioactive compounds, terpenes, phenolics, phloroglucinols, monoterpenes, alkaloids, phenolics, and fatty acids were utilized with the Boolean operators AND, OR, and NOT. Databases such as PubMed, Web of Science, Science Direct, Scopus, Google Scholar, and Reaxys were used. The initial results for keywords such as ferns and phenolics yielded 13,000 papers, ferns and terpenes yielded 2690 papers, and ferns and phloroglucinols yielded 520 papers. These were manually filtered to select only reports discussing chemistry work, which resulted in 2000, 556, and 43 articles, respectively. Subsequently, only reports that discovered new natural products with significant bioactivity were included to yield a reference number of 167 papers. Regarding the biological activities of ferns, 4070 papers were initially obtained and filtered by removing 3007 articles focusing on chemical synthesis and 850 articles about lycophytes to give a final total of 50 papers.

## 3. Chemical Classes of Compounds Isolated from Ferns

Phloroglucinols are subdivided according to their number of monomers into mono, di, tri, tetra and phlorotanins. As one of the monomeric phloroglucinols, polyprenylated acylphloroglucinols (PPAPs) are endowed with fascinating chemical structures and biological activities as a source of lead compounds because they are unique, and many are still untapped. The search results in the PPAPs databases demonstrated the presence of more than 850 molecules with disparate bioactivities. To mention just a few, antiadipogenic, antidepressant, antiparasitic, antimicrobial, antiviral, leishmanicidal, molluscicide and immunomodulatory effects were noted [13].

### 3.1. Polyketide-Terpenoid Hybrids

#### Dryopteridaceae Family

The two genera *Elaphoglossum* and *Dryopteris* contained all the phloroglucinol derivatives isolated before and reported; for instance, there were C-glycosides as dryopteroside from *Dryopteris crassirhizoma* Nakai [14], trisflavaspidic acid ABB and BBB, flavaspidic acid AB and PB, filixcic acid ABA and PBP, dryocrassin, and albaspidin AA [14]. Others included elaphopilosins A-E and elaphogayanin A-C [15] and lindbergins A-D and E-I [16]

PPAPs serve as chemotaxonomic and phylogenetic markers of the genera *Dryopteris* and *Elaphoglossum*, being of significant abundance in this type of molecule [17]. Two new acyl phloroglucinol dimer derivatives, namely, paleacenins A **1** and B **2** with filicinic acid geranylated moieties, were isolated from the nonpolar fractions of the fern *Elaphoglossum paleaceum* (Hook. & Grev.) Sledge. Paleacenin A was a combined filicinic acid and phloroglucinol ring separated by a central methylene. The prenyl side chain attached to C-4 of the filicinic acid was related to the known yungesins. Furthermore, the acetate group at C-6 was verified through long-range coupling between OH-5 and C-19 as well as the carbonyl at C-1, thus establishing the β-triketone system with its keto enol tautomerisms, which explained the chelation and extra deshielding of the acetate group nuclei. While paleacenin A was active as an inhibitor of MAO-A and MAO-B with IC_50_ values of 31 and 4.7 μM, respectively, and more selectivity towards MAO-A, paleacenin B revealed IC_50_ values of 1.3 and 4.4 μM with selectivity towards MAO-B [18].

These compounds were unique in possessing the geranylated filicinic acid moiety and were only preceded by the yungensins obtained from *Elaphoglossum yungense* de la Sota [15]. It is notable that antimicrobial assays and biofilm inhibition might yield promising results, and researchers are encouraged to carry out these investigations based on the reported activities of yungensins and their structural similarity with the paleacenin compounds.

**Figure 1:**
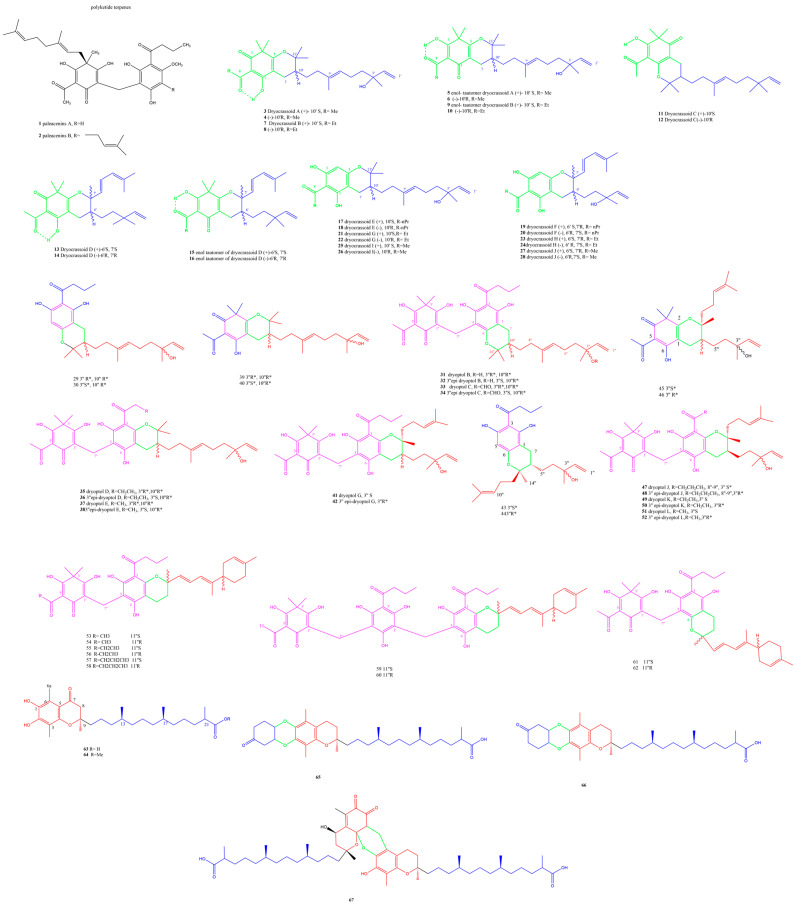
Polyketide-terpene compounds **1-67** isolated from ferns (2017–2023). The two chiral centers have the same configuration, denoted as R*. The two chiral centers have opposite configurations, denoted as S*.

Dryocrassoids (A-J) **3-28** (Figure 1), unique acylphloroglucinol meroterpenoid enantiomeric pairs based on nerolidol-type sesquiterpenes, were isolated from *Dryopteris crassirhizoma* Nakai with six other known phenolic compounds and recorded moderate to low antiviral activity against HSV-1. The structures were assigned based on 1D, 2D, HRMS, and ECD and compared to (±)-hyperjaponol A [19,20] and nerolidol. Dryocrassoid A was identified as a racemic keto nol tautomer, and its signal showed two fragments I and II marking an unconventional link between the filicinic acid and a nerolidol sesquiterpene, respectively, which warrants further biosynthetic pathway identification work, proposedly with similarity to hyperjaponol A [19]. Fragment I only differed from filicinic acid in the presence of an acetyl group instead of the isobutyl and was located at C-2. The linkage was established between the two fragments based on Correlation Spectroscopy (COSY) and Heteronuclear Multiple Bond Correlation (HMBC) that verified the C-7–C-10′ bond. Furthermore, C-5 and C-11′ were shifted down field, revealing the presence of an oxo-bridge that formed a pyran ring. The amounts of the discovered isomers were minute, between 6 and 32 mg, which further encourages biotechnological interventions to enhance the production of these compounds [20].

The unique meroterpenoidal acylphloroglucinols, with the parent name dryoptol, were isolated from *Dryopteris crassirhizoma* Nakai as either mono or dimeric skeletons fused at C-10″/C11″ (type-I) or C-6″/C-7″ (type-II) to the nerolidiol moiety **67-90** (Figure 1). (±)-Dryoptols B-E and their (±)-3″-epi-dryoptols B, C, D, and E **31-38**; (±)-3″-epi-dryoptol G; (±)-dryoptol G **41-42**; and (±)-dryoptols J-L and their 3″epimers **47-52** were isolated as racemes, and the crystallographic data were deposited for (±)-dryoptol A 29 and its 3″-epi isomer **30** and its NMR compared to dryocrassoid E as a reference compound. Dryoptol isomers featured the first structurally mixed molecules between mono acylphloroglucinols and dehydro-theonelline with a pronounced antifungal activity of 1.61 µg/mL for dryoptol D and its epimer compared to fluconazole, whose MIC value is 3.41 µg/mL [21]. Comparing the assignments on the left of C-3″ manifested that dryoptols G, H, and I were shielded compared to the respective signals in their epi-3″ dryoptols G, H, and I, and assignments on the right of C-3″ in dryoptols G, H, and I were all deshielded compared to their C-3″ epimers epi-3″ dryoptols G, H, and I, suggesting the structural correlation of the six compounds [21].

The meroterpenoids dryoptins and 11″ epi dryoptins A-E **53-62** were isolated from the same fern, revealing a new possibility of the combination of dimeric and trimeric phloroglucinols with dehydrotheonelline with a marked antifungal activity against standard *Candida albicans* with an MIC value of 1.61 µg/mL compared to fluconazole (FLC): 3.41 µg/mL [22].

The genus *Dryopteris* was characterized mainly by phloroglucinol derivatives [23,24,25] with few reports on phenolic constituents. Novel tocopherol skeletons were decorated and identified in *Dryopteris crassirhizoma* Nakai. For example, drycrasspherol A **63** featured a 1,2,4 tri oxosubstituted benzene ring combined with a 13′-carboxy-*α*-tocopherol side chain with RRS absolute configurations in the 9, 13, 17-positions, similar to previously isolated tocopherols. The C-22 methoxy isomer was isolated and named drycrasspherol B **64** (Figure 1). Drycrasspherol C **65** (Figure 1) was composed partially of dryocrasspherol A with two more degrees of unsaturation to form rings C and D; moreover, it showed a resemblance to the known compound 3,4-dihydroxy-4-methylcyclohexanone. Compounds **65-67** (Figure 1) represented the new 6/6/6/6 (A/B/C/D) ring system with the same stereochemistry at C-9, C-13, and C-17 based on their common biosynthetic pathway. Drycrasspherol E **67** signified a unique dimer of pentacyclic structure. Correlations between the ring E methylene protons were asserted with C-6, C-5, and C-1 or C-2′, C-3′, and C-4′ in each of the similar monomers of ring B, and the typical Diels–Alder reaction was suggested to be the mechanism of dimerization. Only drycrasspherol A exhibited a promising antiviral effect against respiratory syncytial virus with an IC_50_ value of 6.50 μM compared to ribavarin [26].

### 3.2. Polyketides

#### 3.2.1. Dryopteridaceae Family

**Figure 2:**
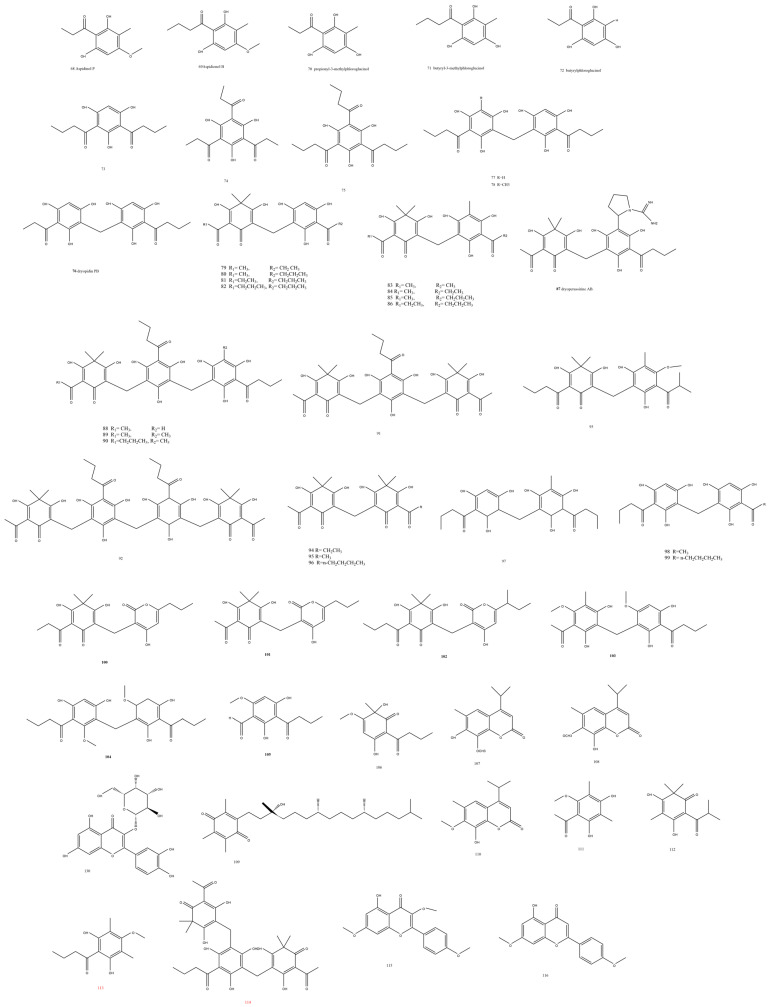
Polyketide compounds **68-116** isolated from ferns (2017–2023).

Five propionyl and butyryl acylated monomeric phloroglucinol derivatives **68-72** were isolated from the dichloromethane fraction of *Dryopteris crassirhizoma* Nakai [27]. Upon studying the antiplatelet activity, structure–activity relationships concluded that three key features in the phloroglucinol skeleton were necessary for the antiplatelet effect: the C-1 butanonyl group, the C-4 hydroxy group, and the C-3 methyl group [27]. Compounds **68** and **69** (Figure 2) were reported to have antimicrobial activity (2 µg/mL) against MRSA that was very comparable to vancomycin both in vitro and in vivo, where virulence factor suppression and ribosomal inhibition played major roles [28].

*Dryopteris crassirhizoma* Nakai rhizome methanolic extract was the source of about 20 phloroglucinol derivatives **73-92** (Figure 2), with monomeric, dimeric, trimeric, and tetrameric types whose protein tyrosine phosphatase 1B enzyme (PTP1B) inhibition was measured from the dimeric flavaspidic acids AB and PB and norflavaspidic acid PB to the trimeric antiviral filixic acid ABA or the tetrameric types as dryocrassin ABBA [29]. The new molecules were **76** and **87**. While dryopidin PB **76** (Figure 2) was identified by comparison to the known methylene-bis-phlorobutyrophenon, as the former manifested a propyl group instead of a methyl group in the latter, dryopcrassirine AB **87** (Figure 2) revealed a unique phloroglucinol structure with a pyrrolidine-1-carboximidamide group confirmed by the MS/MS analysis together with the HMQC and HMBC correlations to the quaternary carbons in the butyrylated phloroglucinol ring. Signals of the pyrrolidine ring were further correlated to the imine functionality by HMBC. Both compounds had a moderate to low PTP1B inhibitory effect compared to ursolic acid [29]. The former was compared to the monomeric known phlorobutyrophenone whose carbon number and obtained molecular formula did not match but suggested the possibility of its dimeric structure. The weakest enzyme inhibition, IC_50_ from 45 to >100 μM, was recorded for monomeric phloroglucinols **73** and **74**, followed by the dimeric compounds with IC_50_ values from 6.0 to 41 μM with a strong emphasis on the nature of the side chain chemical structures, as it could alter the enzyme sensitivity as in **75**, whose IC_50_ value was 14.3 μM despite being a monomer. Propyl, acetyl, and butyryl side chains resulted in significant activity as in flavaspidic acid PB **86**, flavaspidic acid AB **85**, and norflavaspidic acid PB **81**, respectively [29].

Disaspidin BB **93**, a pseudoaspidinol derivative attached to a phloroglucinol ring [30], was isolated from *Dryopteris fragrans* (L.) Schott and revealed promising topical antimicrobial effects against *S. haemolyticus* (SHA), Staphylococcus epidermidis (SEP), and methicillin-resistant *S. aureus* (MRSA); moreover, various ceftazidime-resistant strains were sensitive to **93** with MIC values of 1.67–2.71 μg/mL. Furthermore, it showed a biofilm scavenging effect on SEP [31,32]. The study is a continuation of Teng’s work [30], which outlined a SAR correlation for pseudo aspidinols, particularly the C-4 butyryl, C-6, and the phloroglucinol hydroxyl groups.

Six known phenolic compounds **94-99** from the fronds’ exudate of *Dryopteris crassirhizoma* Nakai exhibited moderate to low antiviral activity against HSV-1 [20,33]. The compounds represented the albaspidin and araspidin nuclei with only acyl group variations in the dimeric filicinic acid units linked with a methylene group.

More acylphloroglucinols with a structural similarity to phloropyron BB were obtained from *Dryopteris championii* (Benth.) C. Chr. in small amounts of between 3 and 11 mg. Prominent features that aided in the identification were the length of the aliphatic chain next to the carbonyl C-3, which varied from methyl, ethyl, or propyl in phloropyrons B and A **100-101** (Figure 2) compared to phloropyron BB as manifested by the H-H COSY between H-9 and H-10 and the HMBC between H–C10 and C8. Moreover, the pyronone attached chain was isobutyl in phloropyron C **102** (Figure 2). Another two compounds showed analogy to margaspidin BB and were characterized as the C-4 methoxy derivative with a methyl group instead of the carbonyl attached propyl group, named margaspidin A **103** (Figure 2), and margaspidin B **104** (Figure 2) with two additional methylenes and the absence of the methyl group. Only phloropyron A and pseudoaspidinol A **105** (Figure 2) showed moderate bactericidal activity against *Staphylococcus aureus*, *Escherichia coli*, *Bacillus subtilis*, and *Dickeya zeae*; this was particularly the case for *Bacillus*, where the two compounds displayed MIC values of 16 μg/mL [34].

Dryofragone **106** and dryofracoumarin B **107** (Figure 2) were isolated and identified from the petroleum ether fraction of the fragrant woodfern *Dracaena fragrans* (L.) Schott methanolic extract; furthermore, six known compounds **108-113** were isolated by means of cytotoxicity-guided separation using the MTT and CCK-8 assays [35]. Dryofragone was an acylphloroglucinol enantiomeric pair with a cyclohexadiene ring hypothesized based on the demonstrated long-range coupling between H-4/C-2, C-3, C-5, and C-6 as well as between CH_3_-11/C-1, C-2, and C-3. A butyryl group was attached to C-6 as manifested by the HMBC correlations from C-8 protons to carbons 6, 7, 8, and 9 [35]. A strong in vitro antiviral effect against H5N1 neuraminidase was revealed by filixic acid ABA **114** and dryocrassin ABBA with IC_50_ values of 29.5 and 18.5 μM, respectively [23]. Similarly, **114** demonstrated an anti-influenza effect against the A/Puerto Rico/8/1934 (H1N1) with an IC_50_ of 38 μg/mL compared to ribavirin, whose IC_50_ was 40 μg/mL [21].

Methoxylated flavonoids **115-124** were obtained from *Asplenium anceps* and demonstrated an antitumor effect against A549, MCF-7, and HL-7702 with a moderate IC_50_ range between 16 and 85 μM [36].

#### 3.2.2. Pteridaceae Family

**Figure 3:**
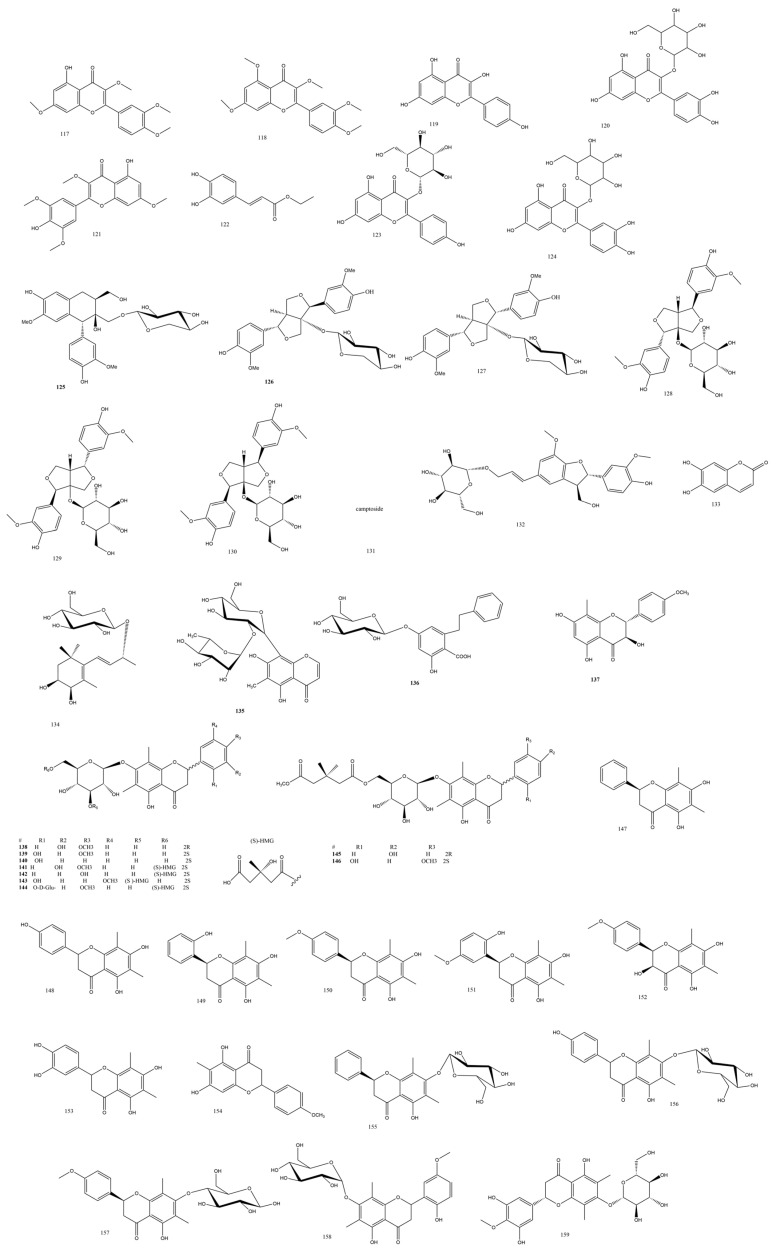
Polyketide compounds **117-159** isolated from ferns (2017–2023).

Three new lignans **125-127** (Figure 3) were identified from *Pteris laeta* Wall. as analogs of 8′-hydroxy-(+)-isolariciresinol-9-*O*-*β*-*D*-xylopyranoside. While **125** was denoted as having a hydroxy group in the 8 position instead of 8′, **126** and **127** were syringaresinol derivatives revealing a xylose sugar moiety instead of glucose in the former and a 7R, 8S, 7′S, 8′R stereochemistry in **127** as manifested by comparing the experimental ECD to the calculated one [37]. Lignans **128-130** were not tested for their biological activity.

#### 3.2.3. Aspleniaceae Family

Interestingly, the same fern was reported to contain iridoid glycosides such as camptoside **131** (Figure 3), which was detected with a unique C-C bond connecting a 4-hydroxystyryl group to its C-6 position [38]. Although it differed from the known methyl ester of E-6*-O-p*-coumaroyl scandoside by only *m*/*z* 44, possibly interpreted as liberated CO_2_ during the MS fragmentation, camptoside was not detected upon heating the E-6*-O-p*-coumaroyl scandoside and re-running the same experiment again, which proved its natural origin. This unusual iridoid with other known compounds **131-133** (Figure 3) revealed a potent NO production inhibitory effect in RAW 264.7 macrophage cells of 11.2 μM compared to the curcumin standard of 10.1 μM.

#### 3.2.4. Onocleaceae Family

The genus *Matteuccia* is composed of five species, and most of the phytochemical work was carried out on three of them: *Matteuccia orientalis* (HOOK.), *Matteuccia Intermedia* C. Chr., and *Matteuccia struthiopteris* (L.) Tod. [39]. The alcoholic rhizome extract of *Matteuccia orientalis* (HOOK.) (Onocleaceae) yielded two new phenolic compounds, matteucens I **135** and J **136** (Figure 3). Matteucen I was a C-*β*-*D*-glucoside connected to a phenyl chromone aglycone moiety. Matteucen II was a bibenzyl phenolic compound analogous to the known molecule 5-*β*-*D*-glucosyloxy-3-hydroxy-trans-stilbene-2-carboxylic acid, yet it is lacking the trans-olefins and instead possesses two methylenes between the two benzenes. Although these two compounds did not have any reports of their biological activities, many pharmacological effects were recorded for *Matteuccia* sp.; for instance, antidiabetic, antioxidative, and anti-inflammatory effects were reported [40]. The methyl group attached to ring A was inserted in the fern’s biosynthetic reaction pattern; thus, several C-methylated flavanones have been reported from the genus *Matteuccia* with different ring B oxygenation combinations [41].

The C-methylated ring A flavanonol demethyl matteucinol **137** was obtained from *Matteuccia Intermedia* C. Chr. [42] together with the C-methylated flavanone glucosides **137-145** (Figure 3) and twenty known compounds **146-167** all presenting C-methylation in ring A at the C-8 position. The SAR correlations suggested the importance of the 7-OH group as a crucial substitution for *α*-glucosidase inhibitory activity, which agreed with the previous results of Cao et al. [43]. Moreover, the combined C-methylation in the 6 and 8 positions in ring A with either or both the hydroxylation or methoxylation of ring B added more to the *α*-glucosidase inhibitory effect [39]. Compounds **149-160** and **162-170** were either inactive as *α*-glucosidase inhibitors or were not assayed at all due to insufficient amounts [44].

The extracts from two ferns, *Matteuccia orientalis* (Hook.) Trevis and *M*. *struthiopteris* (L.) Tod., which are widely used in Chinese cuisine, exhibited reduced IL-1β gene expression in a dose-dependent manner in RAW264.7 cells compared to LPS [45], possibly based on their unique phenolic content, which was found to be increased under the influence of light and stress responses [46].

#### 3.2.5. Tectariaceae Family

The phenolics detected from *Tectaria coadunata* (J.Sm.) C.Chr., a widely traditionally used ethnobotanical pteridophyte in the family *Tectariaceae*, were A-type procyanidins with different polymerization rates, namely, eriodictyol-7-*O*-glucuronide **171** and luteolin-7-*O*-glucoronide **172**, which demonstrated inhibitory bioactivity against several enzymes such as tyrosinases, *α*-amylase, *α*-glucosidase, and acetyl or butyryl cholinesterase. A moderate activity against the BxPC3 cancer cell line was also reported [47].

#### 3.2.6. Dryopteridaceae Family

**Figure 4:**
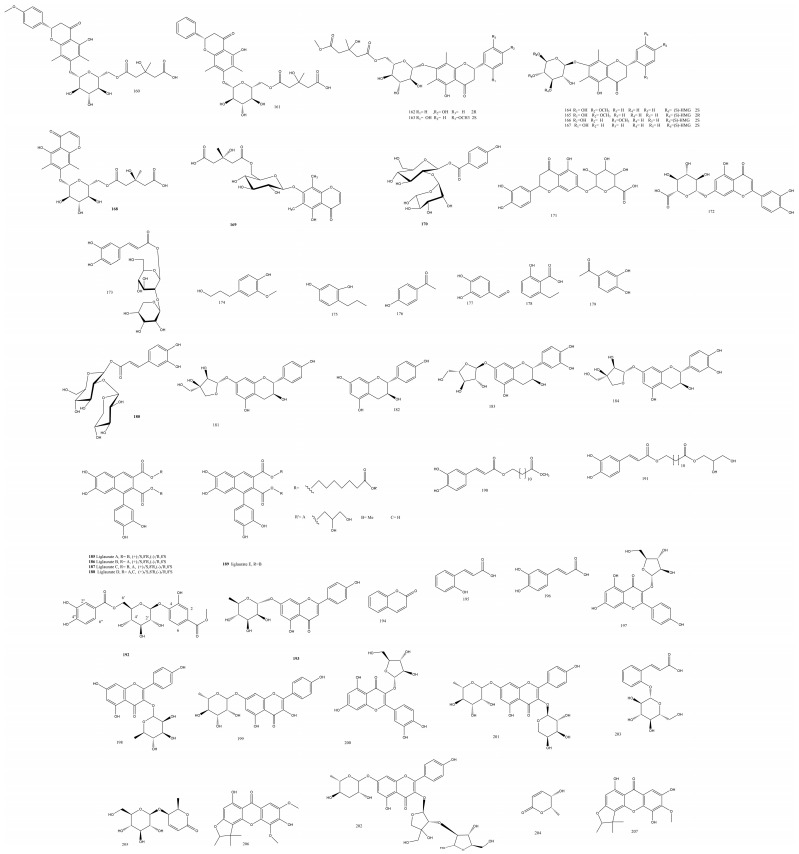
Polyketide compounds **160-207** isolated from ferns (2017–2023).

Eight phenolics with a novel phenyl propanoid fragranoside B **173** (Figure 4) were isolated from *Dracaena fragrans* (L.) Schott, with known phenolic acids **174-179** and only **173** and **179** demonstrating a remarkable anticancer effect against MCF7 cells [48].

#### 3.2.7. Polypodiaceae Family

A few studies were conducted on the Polish traditional fern *Polypodium vulgare* L. revealing its phytochemical components as mainly ecdysteroids, flavonoids, and flavan-3ol derivatives [49]. The rhizomes yielded (+)-afzelechin-7-*O*-*α*-*L*-arabinofuranoside **180** upon water extraction, Moreover, (+)-afzelechin-7*-O-β*-*D*-apiofuranoside **181** and the aglycone (+)-afzelechin **182** with the known flavan-3-ol glycosides (+)-catechin-7-*O*-*α*-*L*-arabinofuranoside **183** and (+)-catechin-7-*O*-*β*-*D*-apiofuranoside **184** were also isolated. Very few studies investigated the biological applications of afzelechin/epiafzelechin; for instance, they were reported to have antioxidant and anti-fresh cut browning effects [1]. The polypody rhizome was also a powerful substance in folk medicine to treat kidney-related ailments. Afzelchins or flavan-3-ols were rarely isolated from ferns; for instance, monomers, dimers, and trimers of the afzelchin glucopyranoside and allopyranosides were isolated from the rhizome of *Drynaria fortune* (Kunze) J. Sm. [50], while monomers and trimers of afzelchin/epiafzechin stereoisomers linked by A- and B-type linkages were isolated from *Selliguea feei* (Bory.) [51].

The rhizomes of the epiphytic fern *Drynaria roosii* Nakaike were extracted to yield five liglaurates A-E **185-189** (Figure 4), the novel bis (lauric acid-12-yl) lignanoates, and the methyl and glyceryl 12-caffeoyloxylaurates. While liglaurates A and B were obtained as pure enantiomers after oxidative coupling reaction, liglaurates C and D were racemic mixtures, and the anticancer effect measured against Hela cells was found to have IC_50_ values of 0.11, 0.24, 0.02, 0.13, 0.34, and 0.17 μM for liglaurates (+)-A, (-)-A, (+)-B, (-)-B, (±)-C, and (±)-D, respectively [52].

The isolated liglaurates were highly analogous to the known epiphyllic acid. These were composed of a lignan skeleton and two methyl 12-hydroxyllaurate, which form a diester between the C9, C9′, and 12″ carboxylic groups. Due to the conformational instability of liglaurate A, determining the absolute configuration of C-7 and C-8 as 7S,8R, was achieved via a synthetic route where the tetra-*O*-methyl derivatives were prepared by reaction with iodomethane followed by sodium hydroxide, which was further confirmed by HRMS [52]. A novel protocatechuic methyl ester **192** (Figure 4) was isolated from *Phymatopteris hastata* (Thunb.) Pic. Serm. comprising two protocatechuic units linked via an ether link from the C-4 to the anomeric carbon in a central glucose unit and between the C-6 of glucose and the C-7′ of the second protocatechuate [53].

The new naringenin glycoside **193** (Figure 4) was confirmed by 1D NMR to be dihydro-flavone, which gave zero optical rotation indicating the possibility of having a racemic mixture. The *α*-glucosidase inhibition activity was assessed for **192** and **194-203**, and they showed potent effects with IC_50_ values ranging between 99 and 697 μg/mL, compared to the standard acarbose [53].

#### 3.2.8. Marattiaceae Family

The rarely reported compounds from ferns, (-)-epi-osmundalactone **204** and angiopteroside **205**, were isolated from the medium polarity fractions of *Angiopteris helferiana* C. Presl of the family Marattiaceae; their content was measured and quantified by UPLC to be 1.54% and 3.2% in the fleshy fern extract, respectively, with promising anti-obesity and anti-inflammatory effects [54]. Osmundalactone was previously isolated from the fern *Angiopteris caudatiformis* Hieron. and showed insect antifeeding activity [55]. Angiopteroside was isolated exclusively from the genus *Angiopteris* and manifested an activity against HIV-1 reverse transcriptase with an IC_50_ of 0.9 mM compared to the didanosine/ddl control with an IC_50_ of 0.87 mM [56].

#### 3.2.9. Metaxyaceae Family

New methylated xanthones were detected in the fern *Metaxya rostrata* (Kunth) C. Presl. This fern has been largely under investigated, from which nearly only xanthones were separated and characterized [57,58,59]. Compounds **206-208** were isolated from the nonpolar extract of the rootlets of *Metaxya rostrata* (Kunth) C. Presl., and structure–activity relationships were suggested based on their assay results as inhibitors of FOxM1 or the induction of cell cycle arrest in G2/M phase [60]. While a single methylation exerted an effect to induce apoptosis, the unmethylated counterparts were of higher efficacy. Furthermore, the di-methylated 2-deprenyl-rheediaxanthone B (XB) and 2-deprenyl-7-hydroxy-rheediaxanthone B were safe with no cytotoxicity. The lack of methylation in the OH-group at position 7 was favored to inhibit topoisomerase I [60].

#### 3.2.10. Cibotiaceae Family

**Figure 5:**
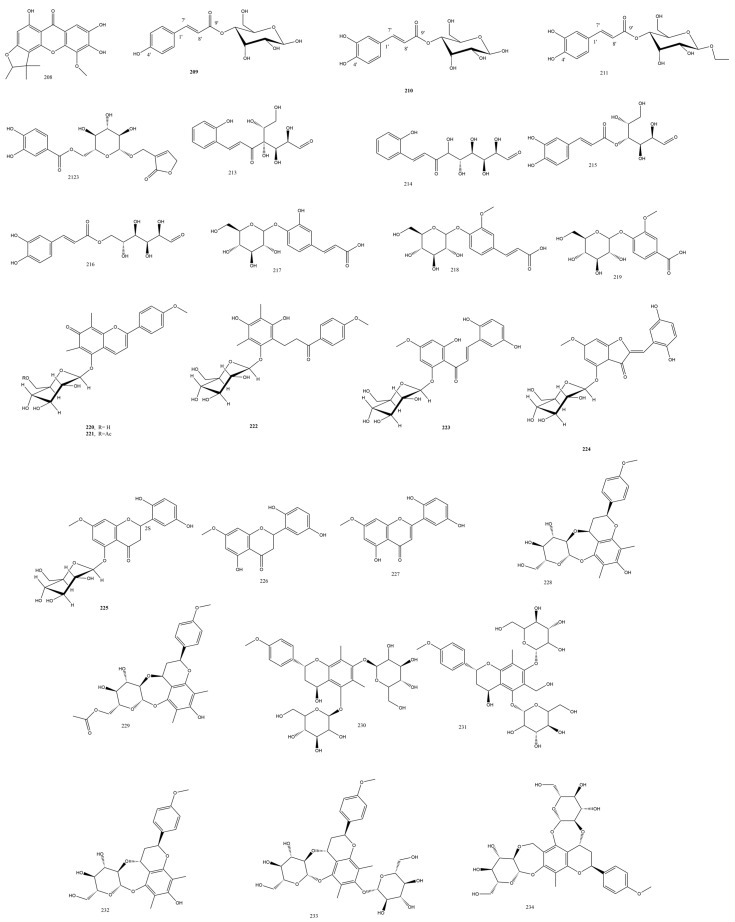
Polyketide compounds **208-234** isolated from ferns (2017–2023).

Substituted glycosides and glycoses were obtained from *Cibotium barometz* (L.) J. Sm. “Gou-ji”, the fern traditionally used for liver and kidney damage. Three new glycosidic compounds, namely, 4-*O*-coumaroyl-*β*-*D*-allose **209** (Figure 5), 4-*O*-caffeoyl-*β*-*D*-allose **210**, and ethyl (4-*O*-caffeoyl)-*β*-*D*-glucopyranoside **211**, were isolated. Phenolic alloses were seldom found in nature, with few reports documenting their chemistry or biological activity. Furthermore, compound **209** significantly exceeded the bicyclol positive control in the hepatoprotective assay against APAP-induced HepG2 cell damage, as it diminished the serum-deprived PC12 cell death and improved viability from 76.4% to 82.9% [42].

#### 3.2.11. Thelypteridaceae Family

Red flavonoids of variant nature were isolated in low yield and characterized from *Pronephrium penangianum* (Hook.) Holttum rhizomes, namely, jixueqisus A-F **220-225** (Figure 5). Jixueqisus A and B possessed the rare 6,8-dimethyl-2-phenyl-7H-1-benzopyran-7-one extended conjugated system, which explained their rich red color. Jixueqisus B was the acetylated analog of Jixueqisus A. Jixueqisus C was comparable to the known abacopterin L except for the lack of the acetyl group. Jixueqisus D was a typical chalcone with four spin systems, the ABX system in the B ring of chalcone, the meta coupled aromatic protons, and the saccharide unit protons; additionally, it had trans-olefinic protons with large J-values. Jixueqisus E shared many features with the D analog, such as the ABX spin protons and the meta coupled protons in ring A, yet the HMBC correlations manifested the presence of an aurone moiety. Cytotoxicity was assayed against the cell lines MCF-7, BGC-823, HepG2, and HCT-116, yet the jixueqisus congeners had negligible activity using paclitaxel as a control [61]. Jixueqisus red pigments were proposed to be generated biosynthetically from **220** through acidification, hydration, tautomerism, hydrogenation, and acetylation processes; furthermore, the heterocyclic ring found in all jixueqisus compounds was anticipated to originate from Michel nucleophilic attack between an unsaturated ketone and a phenol [62]. Flavonoids isolated from the same fern were mostly inactive except for **226** and **228-229**, which were reported to have hepatoprotective or cytotoxic effects [61] (Figure 5).

### 3.3. Steroids and Fatty Acids

#### Athyriaceae Family

**Figure 6:**
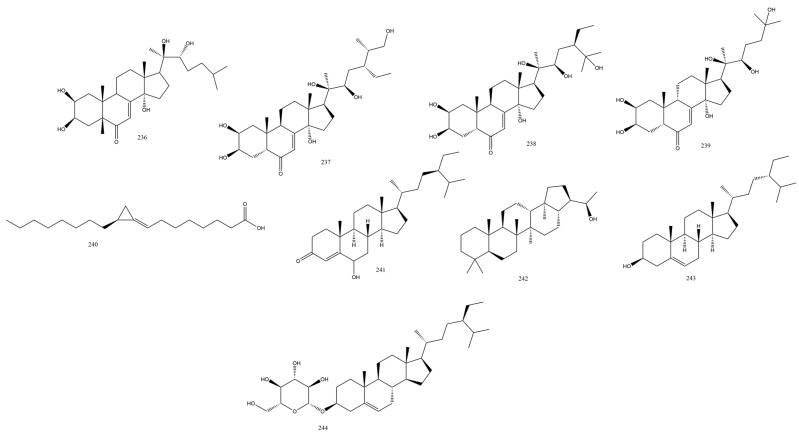
Fatty acid and steroidal compounds **236-244** isolated from ferns (2017–2023).

For the first time from ferns, (2R)-3-(4′-hydroxyphenyl) lactic acid **236** was isolated and identified in the fronds of *Diplazium esculentum* (Retz.) Sw. together with ecdysteroids as ponastrone A, amarasterone A1, and makisterone C, **235-238**. *Diplazium esculentum* (Retz.) Sw. fronds are eaten as vegetables in many countries and are used in folk medicine as a treatment for diabetes and topically for scabies and boils [63]. The stereochemistry of **236** was based on the optical rotation values measured and by comparing them to published values of the R and S enantiomers [64]. Ecdysteroids, known as insect hormones, are polyhydroxy steroids new to the genus *Diplazium* and deserve additional study regarding their chemo taxonomical significance. The Amarasterone A1 stereochemistry was assigned as the (24R, 25S)-isomer based on the superimposition of its NMR data with that previously isolated and confirmed by the synthesis of the molecule from *Leuzea carthamoides* Willd. (Asteraceae) [65] (Figure 6).

*β*-Ecdysterone is the typical 20-hydroxyecdysone, which was revealed to have a promising bone proliferative effect via the BMP2/Smad/Runx2 signaling pathway [36]. Despite the utility of forming the 7,8-dihydro analog based on their wide biological activity, this was sterically hindered in ecdysteroids with their *γ*-hydroxy-*α*, *β*-enone group.

The number of fatty acids with incorporated cyclopropane rings is scarce in nature. A few examples mentioned here from plants, sponges, and bacteria include sterculinic acid isolated from the kernel of *Sterculia joetida* L. seeds, whose anticancer effect and interaction with body fat composition were reported [66]. Similarly, malvalic acid was isolated from the cluster mallow *Malva verticillate* L. family (Malvaceae) and other Malva species. Amphimic acids A and B from the sponge *Amphimedon* sp. growing in Australia demonstrated effective inhibitory action on DNA topoisomerase I, and majusculoic acid obtained from the marine cyanobacteria depicted potent antifungal and anticandidal activities (MIC value of 8 µM). From ferns, the novel unsaturated FA associated with a cyclopropane ring, adiantic acid, was obtained from the fern *Adiantum flabellulatum* L. and characterized as (S, E)-7-(2-octylcyclopropylidene) heptanoic acid **240**, along with three known compounds: stigmast-4-en-6-β-ol-3-one **241**, β-sitosterol **243**, and isoadiantol B **242**. Adiantic acid showed promising anti-protein tyrosine phosphatase enzyme activity (anti-PTP1B) with an IC_50_ value of 6.99 μM [67].

### 3.4. Terpenes Family

**Figure 7:**
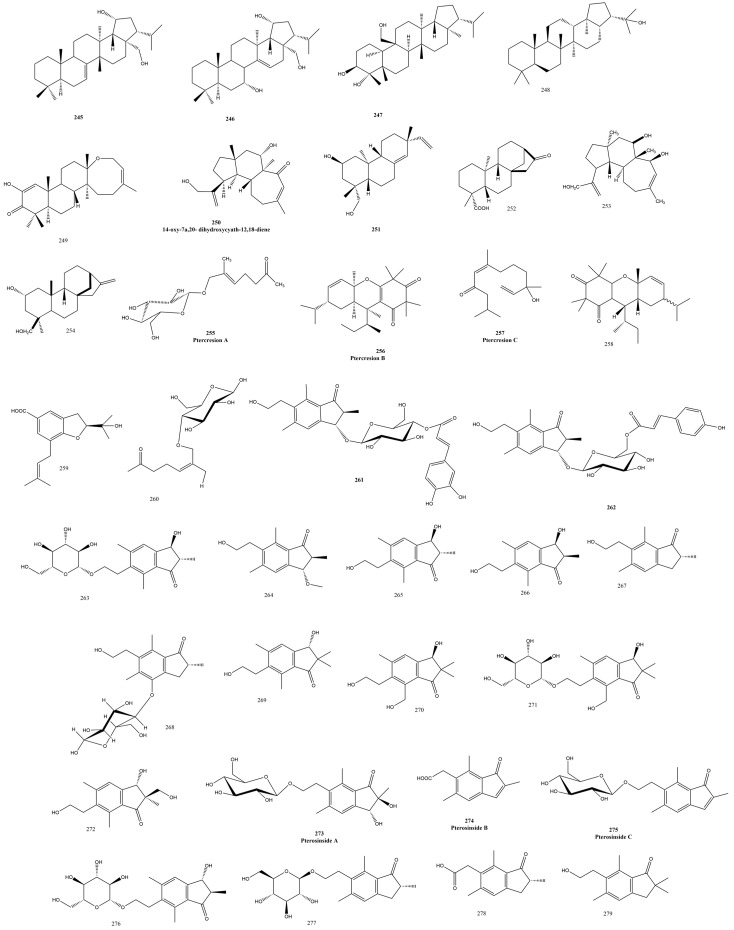
Terpene compounds **245-279** isolated from ferns (2017–2023).

Unlike seed plants, which contain terpene synthases, ferns possess microbial terpene synthase-like (MTPSL) enzymes for terpene production; likewise, fungi, liverworts, hornworts, mosses, and lycophytes contain these enzymes [68] (Figure 7).

#### 3.4.1. Pteridaceae Family

The genus *Adiantum* is composed of 17 species, called the maidenhair fern with several medicinal uses, and about 135 isolated compounds, among them triterpenes, were reported as migrated hopane-, oleanane- [69], and lanostane-type triterpenes [70]. Three hopane-type triterpenes were isolated from the aerial parts of *Adiantum capillus-veneris* Linn. **245-247** (Figure 3 and Figure 4) with significant antifungal effects against *Micrococcus lysodeikticus*, *Alternaria alternata*, and *Helminthosporium maydis*, and their MIC was down into the range of 12.5–3.125 μg/mL [71]. The botanical insecticide 22-hydroxyhopane **248** (Figure 7) was isolated from the fronds of *Adiantum latifolium* Lam., and its trehalase inhibitory and larvicidal activity were recorded since trehalose is a main carbohydrate source in these organisms and its loss contributes to insect mortality [72].

Fern sesterterpenes were reported to assume either a tricyclic cheilanthane or a tetracyclic 18-episcalarane skeleton. The genus *Aleuritopteris* produced sesterterpenes, which were hypothesized to be produced by triterpene synthases in ferns [73]. Although relatively rare in plants, the sesterterpene group of compounds was unveiled in *Aleuritopteris anceps* (Blanf.) Panigrahi, a genus from which only six sesterterpene compounds were detected before with a (17Z)-13, 19-dihydroxylcheilanth-17-ene] moiety: one from *Aleuritopteris Khunii* [74], another from *Aleuritopteris mexicana Fée* [75], and five from *Aleuritopteris krameria* (Franch. & Sav.) Ching [76]. Ancepsone A **249** (Figure 7) was a novel 13, 19-epoxycheilanthane skeleton that was unambiguously characterized with its absolute configuration using single crystal X-ray crystallography. Ancepsone A **249** exhibited a moderate anticancer effect against MCF-7 cell line cells with an IC_50_ value of 12.63 μM as compared to its milder effect against normal HL-7702 cells [36]. *Cheilanthane sesterterpenes* were known to be isolated from a few plants such as Aletris farinose as well as insects and marine sponges [77].

While rarely described from ferns and limited to basidiomycetes and fungi [78], cyathane diterpenes were isolated from *Aleuritopteris albofusca* (Baker) Pic. Serm.; specifically, 14-oxy-7*α*,20-dihydroxycyath-12,18-diene **250** (Figure 7) was isolated, and its structure was comparable to onychiol B, whose abundance and high yield in this fern suggested the possible production of cyathanes by ferns rather than their fungal counterparts. Moreover, ent-8[14],15-pimaradiene-2β,19-diol **251** (Figure 7) was reported and characterized by single-crystal X-ray crystallography [79]. Even though pimaradienes have been reported from plants, they mainly had 3,19-dihydroxy groups; the 2β,19-diol pimaradienes were not common [79]. Ptercresions A **255**, B **256**, and C **257** (Figure 7) were isolated from *Pteris cretica* L., and the latter two compounds showed significant liver-protective effects in paracetamol-induced L02 cells in vitro with EC_50_ values between 107 and 213 μM compared to silymarin [80].

The genus Pteris includes about 300 species, and most of them were used to treat common illnesses in Chinese medicine as *Pteris multifida* Poir. and *Pteris spinosa* (L.) Desv. [80,81]. Illudoid sesquiterpenes, known as pterosins, were recognized as the chemo taxonomical markers of this genus and the family *Pteridaceae*. Pterosins were classified according to their carbon skeleton number into C13, C14, and C15 compounds, which possess several biological activities such as antitumor, anti-inflammatory, and antimicrobial effects [37].

The 1-indanone type sesquiterpenes, which are pterosin compounds, belonging to the illudoid C14 and C15 molecules, were first isolated and characterized from Bracken ferns *Pteridium aquilinum* (L.) Kuhn back in 1978. For example, pterosins A, D, K, L, and Z together with their esters and glucosides, were named pterosides. The nor-sesquiterpenes were also reported by the same group as pterosins B, C, E, F, G, I, J, O, and N with their esters and glucosides [82]. Several later derivatives such as (2R)-pterosin P and dehydropterosin B were reported from *Pteris multifida* Poir. [83]. These molecules possessed two chiral centers at the carbon 2 and 3 positions, which formed their cotton effect CD (Circular Dichroism) at 310–370 nm caused by the n-π* transitions of their conjugated ketones [84].

Recently, more pterosin glucosides of (2S,3S) pterosin C were isolated from the roots of *Pteris multifida* Poir (Pteridaceae) as two new derivatives possessing either caffeoyl or coumaroyl moieties attached to the glucose C-4 or C-6 position, respectively. The first C14 sesquiterpene was (2S,3S)-pterosin C3-*O*-*β*-*D*-[4′-(E)]-caffeoyl]-glucopyranoside **261** (Figure 7), and the second was (2S,3S)-pterosin C3-*O*-*β*-*D*-[6′-(E)-p-coumaroyl]-glucopyranoside **262** (Figure 7). Both compounds showed positive cotton effects at 320 nm indicating that the C-3 hydroxyl group was pseudo axial. Additionally, the anti-configuration of C-3 was assured from a small coupling constant. It is notable that Kim et al. incorrectly referred to the C-3 configuration as trans, which should be anti, as the system was a single bond with free rotation. (2S,3S)-3-Methoxypterosin was reported from nature for the first time after its synthesis by [85]. Furthermore, ten known compounds **263-272** were detected (Table 1). Only compounds **261** and **262** (Figure 7) displayed a significant anticancer effect against the HCT116 human colorectal cancer cell line with IC_50_ values of 8 and 17 μM by increasing the levels of caspase 9 and procaspase 9 [86].

**Figure 8:**
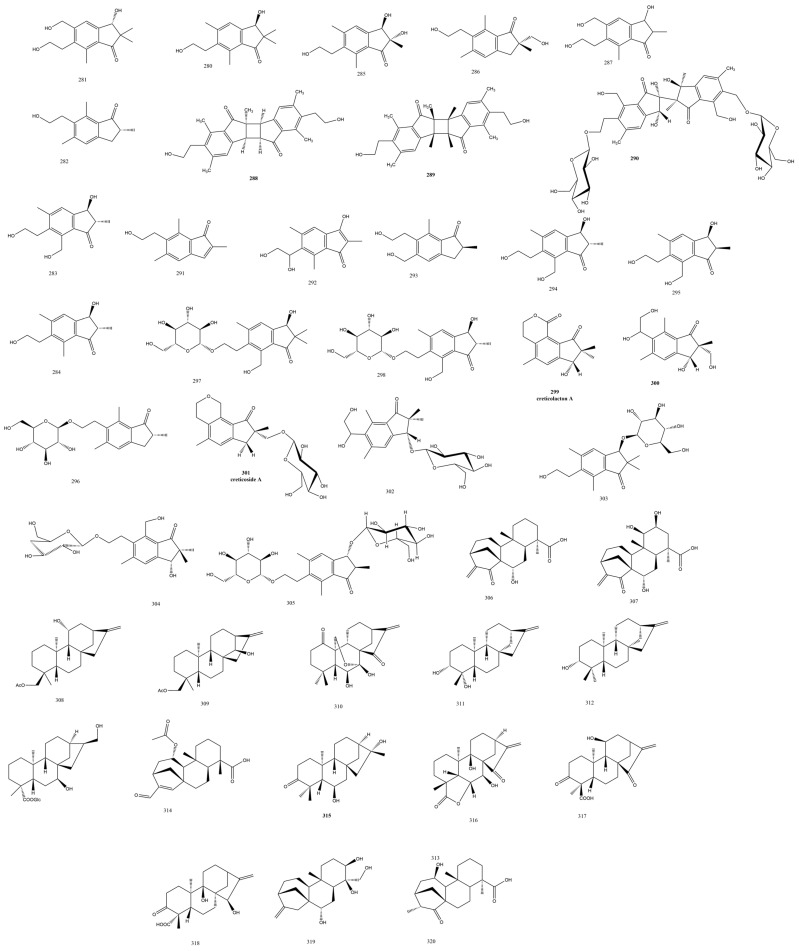
Terpene compounds **280-320** isolated from ferns (2017–2023).

The roots of *Pteris laeta* Wall were the source of about twenty sesquiterpenes **273-288** (Figure 7 and Figure 8) with a powerful neuroprotective activity in Alzheimer disease (AD), as evidenced by in vitro studies involving H_2_O_2_-induced N2a cell damage where the survival rate in all sesquiterpenes ranged from 86 to 91%, with pterosin B exhibiting a neuroprotective effect 200 times that of ginsenoside Rg1 [37]. Although the Pteris species are producers of pterosin toxins, edible pterosin types were identified in this genus as well [87,88,89]. Pterosinsides A, B, and C **273-275** (Figure 7) were isolated and characterized by an olefinic C-2 and C-3 trans glycol system, compared to pterosin E methylene and methylidyne carbons. Zhang et al. inadequately named the sesquiterpene pterosin E compound as peterosin E [84] and the COSY analysis as COZY, which should be pointed out critically here to avoid confusion.

The indanone-skeleton-based sesquiterpenes were isolated from *Pteris obtusiloba* Ching & S.H. Wu. Among the reported bioactivities were antidiabetic, anti-tumor, and antihyperlipidemic effects [90]. Nevertheless, in a unique study considered to be the only one recorded from *Pteris obtusiloba Ching* & S.H. Wu, Peng et al. reported eleven 1H-inden-1-one-stype sesquiterpenes HSs, **288-298** including three novel dimers **288** and **290** (Figure 8) and an isomeric pair **288** and **289**. These skeletons were verified by NMR, 2D NMR, ESI-MS, CD, and X-ray. 1H-Inden-1-one-type sesquiterpenes HSs were also isolated from fungi previously such as *Basoidiomycota*, yet none of them significantly inhibited the *α*-glucosidase enzyme. These results, possibly attributed to experimental errors, warrant more investigation since pterosins and their glycosides were mentioned by more than one study to exhibit promising antidiabetic activity [90,91]. The three obtupterosin dimers A **288-290** revealed IC_50_ values of 27.5 μM, 30.6 μM, and 12.8 μM against the human colon cancer cell line HCT-116 [90,92].

*Pteris cretica* L. aerial parts were the source of the new pterosins, creticolacton A **299** (Figure 8), 13-hydroxy-2(R),3(R)-pterosin L **300**, creticoside A **301** with a 1, 2 glycol side chain attached to C-6 of **300**, and the glycosidic derivative spelosin 3-*O*-*β*-*D*-glucopyranoside **302** (Figure 6). Creticolacton A **299** and **300** (Figure 8) manifested a cytotoxic activity against different human cell lines such as the neuroblastoma cell line SH-SY5Y, gastric cancer cell line SGC-7901, colon cancer cell line HCT-116, and colorectal cancer cell line Lovo, with IC_50_ values of 22.4 μM and 15.8 μM, respectively [93]. It is worth mentioning that the yield was very low from *Pteris cretica* L., as the authors ended up with a few milligrams of each compound although they started with 5 kg of the dried plant and a weight of over one-hundred grams of extract by liquid extraction of n-butanol, petroleum ether, dichloromethane, and ethyl acetate.

Diterpenes of the family *Pteridaceae* were classified into ent-kaurane or ent-kaurenes with hydroxylation mainly encountered in any of the 2, 16, 11, 7, 9, 15, 14, 19, and 12 positions as mono-, bi-, tri-, or tetra-hydroxylation. Epoxidations were seen usually in positions 9, 11, or 16, and oxidation was shown mostly in position 15. Sugar attachments were commonly formed by the hydroxy groups in position 2 or 19, and the type of sugar was primarily glucose or allose [5]. Kaurenes were reported in abundance from plants and fungi [94], and in ferns, the family *Pteridaceae* produced almost all the ent-kaurene or ent-kaurane moieties. Several kaurene and ent-kaurene compounds were isolated in the 95% ethanolic extract of the spike-tooth fern, *Pteris dispar* Kunze. whole plant, among which the 18-acetoxy derivatives **306** and **307** were isolated for the first time and hydroxylations were seen in carbons 17 and 7 [95]; moreover, the isodon 7,20-epoxy-ent-kuarenoid neolaxiflorin L was identified, which was previously isolated from the plant *Isodon excisoides* (Y.Z.Sun) H.Hara, studied for its SAR, and many of its analogs were synthesized to optimize its promising anticancer effects [96]. Neolaxiflorin L **310** manifested a potent cytotoxicity against the four cell lines HCT-116, HepG2, NCI-H1650, and A2780 with an IC_50_ range between 1 and 5 µg/mL [95]. The ent-kaurane-6*β*,16*α*-diol-3-one **315** (Figure 8) was identified from *Pteris ensiformis* Burm. as the first ent-kaurane with an oxidized C-3 position in the Pteris genus and Pterideaceae family [97].

#### 3.4.2. Aspleniaceae Family

Another class of triterpenes was the cycloartane type glycosides characterized by the rare 9*α*,10*α*-cyclopropyl ring; these were isolated from *Asplenium ruprechtii* Sa. Kurata. While **321** and **322** possessed the typical three-membered ring, **323-325** (Figure 8) comprised a new *Asplenium* moiety of 9,19-seco-cycloartane-9,11-en triterpene aglycone in a highly 27 oxidized skeleton of methylene, methylene, or quaternary carbons in the order of 3, 7 (or 23), 24, 25, 30 [98]. **321** and **322** possessed an antitumor activity within 18–60 μM (Table 1).

**Figure 9:**
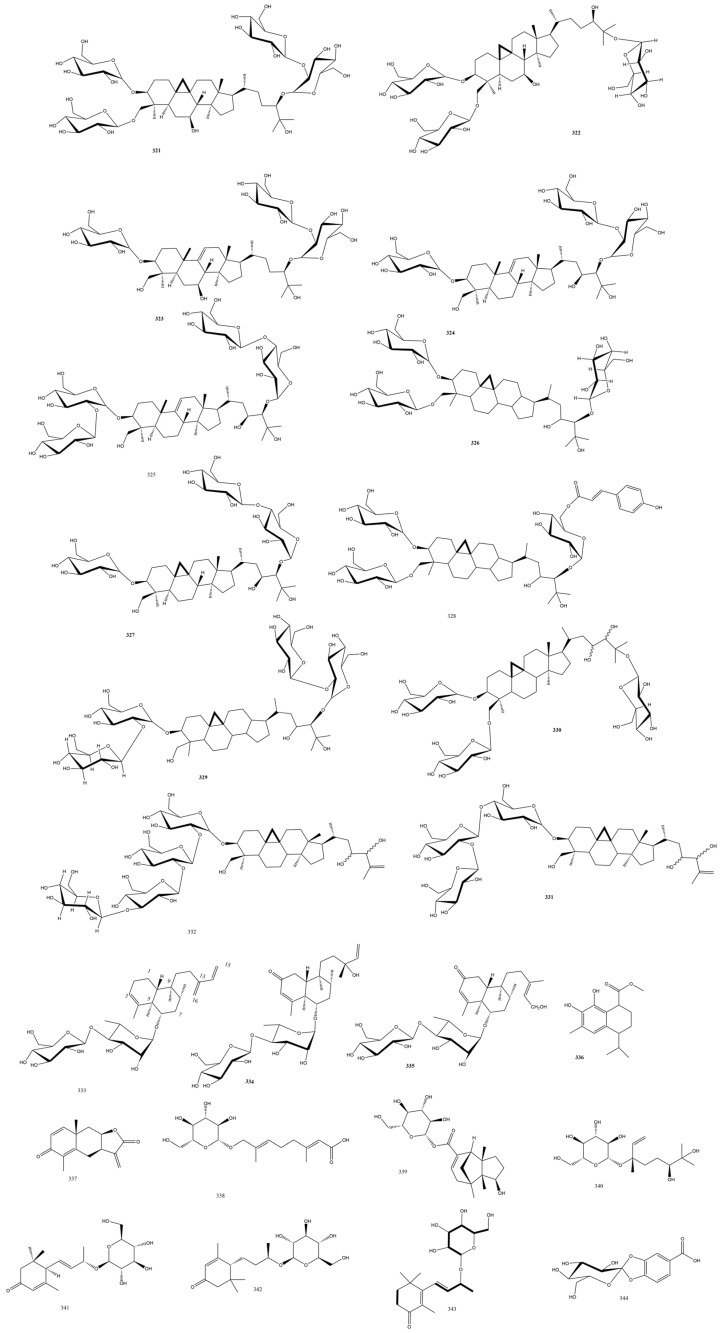
Terpene compounds isolated from ferns (2017–2023).

The cycloartane glycoside aspleniumside A **321** (Figure 9) was isolated from *Asplenium ruprechtii* Sa.Kurata. The relative configuration was assigned using NOESY knowing that CH2-19 in this plant genus is found in the *β*-orientation. Aspleniumside B was comparably assigned to aspleniumside A, being that it only lacked one hexosyl moiety, while aspleniumside C-E belonged to the 9,11-en, 9,19-seco-cycloartane triterpene skeleton. The in vitro assays against the HL-60 and HepG2 cell lines showed significant cytotoxic effects of these compounds with IC_50_ values between 18 and 60 μM [98]. Both the 23, 25-dihydroxycycloartane, aspleniumside F 326 (Figure 9) [99] and the 9, 19-cycloartanes, aspleniumside G **327** [100], as well as aspleniumside H **328** and I **329** were isolated from *Asplenium ruprechtii* Sa.Kurata (Figure 9) but with no appreciable bioactivity. It was notable that these compounds were isolated from the same fern by the same group, and thus, it was better to publish them in the same study. He et al. revealed the isolation of cycloartane triterpenoidal saponins **330-332** (Figure 9) with a triglycosidic nature, which was further confirmed by acid hydrolysis. **333** and **334** were closely related to **330** except for an extra double bond in C-25 or a change in the sugar moieties, respectively. The compounds were obtained in minute yields and may benefit from biotechnological applications to optimize their amounts [101].

**Table 1:** Compilation of isolated compounds, their sources, and bioactivities from ferns.

No.	Chemical Name	Fern Name	Biological Activity	References
1	Paleacenins A	*Elaphoglossum paleaceum* (Hook. & Grev.) *Sledge*	MAO inhibitor	[18]
2	Paleacenins B	*Elaphoglossum paleaceum* (Hook. & Grev.) *Sledge*	MAO inhibitor	[18]
3	Aspidinol P	*Dryopteris crassirhizoma* Nakai	*antimicrobial*	[27]
4	Aspidinol B	*Dryopteris crassirhizoma* Nakai	*antimicrobial*	[27]
5	Propionyl-3-methylphloroglucinol	*Dryopteris crassirhizoma* Nakai		[27]
6	Butyryl-3-methylphloroglucinol	*Dryopteris crassirhizoma* Nakai		[27]
7	Butyrylphloroglucinol	*Dryopteris crassirhizoma* Nakai		[27]
8	1,3-(2,4,6-trihydroxyphenyl)dibutanone	*Dryopteris crassirhizoma* Nakai	*PTP1B inhibitor*	[29]
9	Tripropionylphloroglucinol	*Dryopteris crassirhizoma* Nakai	*PTP1B inhibitor*	[29]
10	Tributyrylphloroglucinol	*Dryopteris crassirhizoma* Nakai	*PTP1B inhibitor*	[29]
11	Dryopidin PB	*Dryopteris crassirhizoma* Nakai	*PTP1B inhibitor*	[29]
12	Methylene-bis-phlorobutyrophenone	*Dryopteris crassirhizoma* Nakai	*PTP1B inhibitor*	[29]
13	Araspidin BB	*Dryopteris crassirhizoma* Nakai		[29]
14	Norflavaspidic acid AP	*Dryopteris crassirhizoma* Nakai		[29]
15	Norflavaspidic acid AB	*Dryopteris crassirhizoma* Nakai	*PTP1B inhibitor*	[29]
16	Norflavaspidic acid PB	*Dryopteris crassirhizoma* Nakai	*PTP1B inhibitor*	[29]
17	Norflavaspidic acid BB	*Dryopteris crassirhizoma* Nakai		[29]
18	Flavaspidic acid AA	*Dryopteris crassirhizoma* Nakai		[29]
19	Flavaspidic acid AP	*Dryopteris crassirhizoma* Nakai		[29]
20	Flavaspidic acid AB	*Dryopteris crassirhizoma* Nakai		[29]
21	Flavaspidic acid PB	*Dryopteris crassirhizoma* Nakai		[29]
22	Dryopcrassirine AB	*Dryopteris crassirhizoma* Nakai		[29]
23	Nortrisflavaspidic acid ABB	*Dryopteris crassirhizoma* Nakai	*PTP1B inhibitor*	[29]
24	Trisflavaspidic acid ABB	*Dryopteris crassirhizoma* Nakai		[29]
25	Trisflavaspidic acid BBB	*Dryopteris crassirhizoma* Nakai		[29]
26	Filixic acid ABA	*Dryopteris crassirhizoma* Nakai	*Antiviral H1N1*	[29]
27	Dryocrassin ABBA	*Dryopteris crassirhizoma* Nakai		[29]
28	Disaspidin BB	*Dryopteris fragrans* (L.) Schott	antimicrobial	[30]
29	(+)-Dryocrassoid A	*Dryopteris crassirhizoma* Nakai	anti-HSV 1 and anti-RSV	[20]
30	(-)-Dryocrassoid A	*Dryopteris crassirhizoma* Nakai	anti-HSV 1 and anti-RSV	[20]
31	Enol-(+)-Dryocrassoid A	*Dryopteris crassirhizoma* Nakai	anti-HSV 1 and anti-RSV	[20]
32	Enol-(-)-Dryocrassoid A	*Dryopteris crassirhizoma* Nakai	anti-HSV 1 and anti-RSV	[20]
33	(+)-Dryocrassoid B	*Dryopteris crassirhizoma* Nakai	anti-HSV 1 and anti-RSV	[20]
34	(-)-Dryocrassoid B	*Dryopteris crassirhizoma* Nakai	anti-HSV 1 and anti-RSV	[20]
35	Enol(+)-Dryocrassoid B	*Dryopteris crassirhizoma* Nakai	anti-HSV 1 and anti-RSV	[20]
36	Enol(-)-Dryocrassoid B	*Dryopteris crassirhizoma* Nakai	anti-HSV 1 and anti-RSV	[20]
37	(+)-Dryocrassoid C	*Dryopteris crassirhizoma* Nakai	anti-HSV 1 and anti-RSV	[20]
38	(-)-Dryocrassoid C	*Dryopteris crassirhizoma* Nakai	anti-HSV 1 and anti-RSV	[20]
39	(+)-Dryocrassoid D	*Dryopteris crassirhizoma* Nakai	anti-HSV 1 and anti-RSV	[20]
40	(-)-Dryocrassoid D	*Dryopteris crassirhizoma* Nakai	anti-HSV 1 and anti-RSV	[20]
41	Enol(+)-Dryocrassoid D	*Dryopteris crassirhizoma* Nakai	anti-HSV 1 and anti-RSV	[20]
42	Enol(-)-Dryocrassoid D	*Dryopteris crassirhizoma* Nakai	anti-HSV 1 and anti-RSV	[20]
43	(+)-Dryocrassoid E	*Dryopteris crassirhizoma* Nakai	anti-HSV 1 and anti-RSV	[20]
44	(-)-Dryocrassoid E	*Dryopteris crassirhizoma* Nakai	anti-HSV 1 and anti-RSV	[20]
45	(+)-Dryocrassoid F	*Dryopteris crassirhizoma* Nakai	anti-HSV 1 and anti-RSV	[20]
46	(-)-Dryocrassoid F	*Dryopteris crassirhizoma* Nakai	anti-HSV 1 and anti-RSV	[20]
47	(+)-Dryocrassoid G	*Dryopteris crassirhizoma* Nakai	anti-HSV 1 and anti-RSV	[20]
48	(-)-Dryocrassoid G	*Dryopteris crassirhizoma* Nakai	anti-HSV 1 and anti-RSV	[20]
49	(+)-Dryocrassoid H	*Dryopteris crassirhizoma* Nakai	anti-HSV 1 and anti-RSV	[20]
50	(-)-Dryocrassoid H	*Dryopteris crassirhizoma* Nakai	anti-HSV 1 and anti-RSV	[20]
51	(+)-Dryocrassoid I	*Dryopteris crassirhizoma* Nakai	anti-HSV 1 and anti-RSV	[20]
52	(-)-Dryocrassoid I	*Dryopteris crassirhizoma* Nakai	anti-HSV 1 and anti-RSV	[20]
53	(+)-Dryocrassoid J	*Dryopteris crassirhizoma* Nakai	anti-HSV 1 and anti-RSV	[20]
54	(-)-Dryocrassoid J	*Dryopteris crassirhizoma* Nakai	anti-HSV 1 and anti-RSV	[20]
55	Albaspidin AP	*Dryopteris crassirhizoma* Nakai		[20]
56	Albaspidin AA	*Dryopteris crassirhizoma* Nakai	anti-HSV 1 and anti-RSV	[20]
57	Albaspidin AB	*Dryopteris crassirhizoma* Nakai	anti-HSV 1 and anti-RSV	[20]
58	Araspindin BB	*Dryopteris crassirhizoma* Nakai	anti-HSV 1 and anti-RSV	[20]
59	1-[3-[(3-acetyl-2,4,6-trihydroxyphenyl)methyl]-2,4,6-trihydroxyphenyl] butanone	*Dryopteris crassirhizoma* Nakai	anti-HSV 1 and anti-RSV	[20]
60	Methylene-bis-phlorobutyrophenone	*Dryopteris crassirhizoma* Nakai	anti-HSV 1 and anti-RSV	[20]
61	Phloropyron A	*Dryopteris championii* (Benth.) C. Chr.	antibacterial	[34]
62	Phloropyron B	*Dryopteris championii* (Benth.) C. Chr.	bactericidal	[34]
63	Phloropyron C	*Dryopteris championii* (Benth.) C. Chr.		[34]
64	Margaspidin A	*Dryopteris championii* (Benth.) C. Chr.		[34]
65	Margaspidin B	*Dryopteris championii* (Benth.) C. Chr.		[34]
66	Pseudoaspidinol A	*Dryopteris championii* (Benth.) C. Chr.	bactericidal	[34]
67	Dryoptol A	*Dryopteris crassirhizoma* Nakai		[21]
68	3″-Epi-dryoptol A	*Dryopteris crassirhizoma* Nakai	anti-inflammatory	[21]
69	Dryoptol B	*Dryopteris crassirhizoma* Nakai	antifungal+FLC	[21]
70	3″-epi-dryoptol B	*Dryopteris crassirhizoma* Nakai	antifungal+FLC	[21]
71	Dryoptol C	*Dryopteris crassirhizoma* Nakai		[21]
72	3″-Epi-dryoptol C	*Dryopteris crassirhizoma* Nakai		[21]
73	Dryoptol D	*Dryopteris crassirhizoma* Nakai	antifungal+FLC	[21]
74	3″-epi-dryoptol D	*Dryopteris crassirhizoma* Nakai		[21]
75	Dryoptol E	*Dryopteris crassirhizoma* Nakai	antifungal+FLC	[21]
76	3″-Epi-dryoptol E	*Dryopteris crassirhizoma* Nakai	antifungal+FLC	[21]
77	Dryoptol F	*Dryopteris crassirhizoma* Nakai		[21]
78	3”-Epi-dryoptol F	*Dryopteris crassirhizoma* Nakai		[21]
79	Dryoptol G	*Dryopteris crassirhizoma* Nakai	anti-inflammatory	[21]
80	3″-Epi-dryoptol G	*Dryopteris crassirhizoma* Nakai	anti-inflammatory	[21]
81	Dryoptol H	*Dryopteris crassirhizoma* Nakai		[21]
82	3″-Epi-dryoptol H	*Dryopteris crassirhizoma* Nakai		[21]
83	Dryoptol I	*Dryopteris crassirhizoma* Nakai	antifungal+FLC	[21]
84	3″-Epi-dryoptol I	*Dryopteris crassirhizoma* Nakai	antifungal+FLC	[21]
85	Dryoptol J	*Dryopteris crassirhizoma* Nakai		[21]
86	3″-Epi-dryoptol J	*Dryopteris crassirhizoma* Nakai		[21]
87	Dryoptol K	*Dryopteris crassirhizoma* Nakai	anti-inflammatory y, antifungal+FLC	[21]
88	3″-Epi-dryoptol K	*Dryopteris crassirhizoma* Nakai	anti-inflammatory, antifungal+FLC	[21]
89	Dryoptol L	*Dryopteris crassirhizoma* Nakai	antifungal+FLC	[21]
90	3″-Epi-dryoptol L	*Dryopteris crassirhizoma* Nakai	antifungal+FLC	[21]
91	Dryoptin A	*Dryopteris crassirhizoma* Nakai		[23]
92	Epi-dryoptin A	*Dryopteris crassirhizoma* Nakai	antifungal	[23]
93	Dryoptin B	*Dryopteris crassirhizoma* Nakai		[23]
94	Epi-dryoptin B	*Dryopteris crassirhizoma* Nakai		[23]
95	Dryoptin C	*Dryopteris crassirhizoma* Nakai	antifungal	[23]
96	Epi-dryoptin C	*Dryopteris crassirhizoma* Nakai	antifungal	[23]
97	Dryoptin D	*Dryopteris crassirhizoma* Nakai	antifungal	[23]
98	Epi-dryoptin D	*Dryopteris crassirhizoma* Nakai	antifungal	[23]
99	Dryoptin E	*Dryopteris crassirhizoma* Nakai		[23]
100	Epi-dryoptin E	*Dryopteris crassirhizoma* Nakai		[23]
101	Dryofragone	*Dracaena fragrans* (L.) Schott	*Cytotoxic*	[35]
102	Dryofracoumarin B	*Dracaena fragrans* (L.) Schott	*Cytotoxic*	[35]
103	Norflavesone	*Dracaena fragrans* (L.) Schott	*Cytotoxic*	[35]
104	Aspidinol	*Dracaena fragrans* (L.) Schott	*c* *ytotoxic and immunomodulator*	[35]
105	Dryofracoumarin A	*Dracaena fragrans* (L.) Schott	*c* *ytotoxic and immunomodulator*	[35]
106	Vitamin E quinone	*Dracaena fragrans* (L.) Schott	*cy* *totoxic*	[35]
107	Albicanol	*Dracaena fragrans* (L.) Schott	*c* *ytotoxic*	[35]
108	2′,4′-dihydroxy-6′-methoxy-3′,5′-dimethylchalcone	*Dracaena fragrans* (L.) Schott	*c* *ytotoxic*	[35]
109	Fern-7(8)-en-19*α*, 28-diol	*Adiantum capillus-veneris* Linn.	antifungal	[71]
110	3*β*,4*α*,25-trihydroxyfilican	*Adiantum capillus-veneris* Linn.	antifungal	[71]
111	Pteron-14-ene-7*α*,19*α*,28-triol	*Adiantum capillus-veneris* Linn.	antifungal	[71]
112	22-hydroxyhopane	*Adiantum latifolium Lam.*	trehalase inhibitory and larvicidal activity	[72]
113	Ancepsone A	*Aleuritopteris anceps* (Blanf.) Panigrahi	anticancer	[36]
114	5-hydroxy-3,7,4′-trimethoxyflavone	*Aleuritopteris anceps* (Blanf.) Panigrahi	anticancer	[36]
115	*β*-sitosterol	*Aleuritopteris anceps* (Blanf.) Panigrahi	anticancer	[36]
116	5-hydroxy-7,4′-dimethoxyflavone	*Aleuritopteris anceps* (Blanf.) Panigrahi	anticancer	[36]
117	3,7,3′,4′-tetramethyl-quercetin	*Aleuritopteris anceps* (Blanf.) Panigrahi	anticancer	[36]
118	5,4′-dihydroxy-3,7-dimethoxyflavone	*Aleuritopteris anceps* (Blanf.) Panigrahi	anticancer	[36]
119	Kaempferol	*Aleuritopteris anceps* (Blanf.) Panigrahi	anticancer	[36]
120	Daucosterol	*Aleuritopteris anceps* (Blanf.) Panigrahi	anticancer	[36]
121	Quercetin-3-glucopyranoside	*Aleuritopteris anceps* (Blanf.) Panigrahi	anticancer	[36]
122	14-oxy-7*α*,20-dihydroxycyath-12,18-diene	*Aleuritopteris albofusca* (Baker) Pic.Serm		[79]
123	Ent-8[14],15-pimaradiene-2*β*,19-diol	*Aleuritopteris albofusca* (Baker) Pic.Serm		[79]
124	Myricetin 3,7,3′,5′-tetramethyl ether	*Aleuritopteris albofusca* (Baker) Pic.Serm		[79]
125	Ethyl caffeate	*Aleuritopteris albofusca* (Baker) Pic.Serm		[79]
126	Ent-16-oxo-17-norkauran-19-oic acid	*Aleuritopteris albofusca* (Baker) Pic.Serm		[79]
127	Onychiol B	*Aleuritopteris albofusca* (Baker) Pic.Serm		[79]
128	Aleuritopsis B	*Aleuritopteris albofusca* (Baker) Pic.Serm		[79]
129	Astragalin	*Aleuritopteris albofusca* (Baker) Pic.Serm		[79]
130	Hyperin	*Aleuritopteris albofusca* (Baker) Pic.Serm		[79]
131	Ptercresion A	*Pteris cretica* L.		[80]
132	Ptercresion B	*Pteris cretica* L.	hepatoprotective	[80]
133	Ptercresion C	*Pteris cretica* L.	hepatoprotective	[80]
134	Callisalignene D	*Pteris cretica* L.	hepatoprotective	[80]
135	Berberiside A	*Pteris cretica* L.		[80]
136	Creoside I	*Pteris cretica* L.		[80]
137	(2S,3S)-pterosin C 3-*O*-*β*-*D*-[4′-(E)-caffeoyl]-glucopyranoside	*Pteris multifida* Poir.	anticancer	[86]
138	(2S,3S)-pterosin C 3-*O*-*β*-*D*-[6′-(E)-*P*-coumaroyl]-glucopyranoside	*Pteris multifida* Poir.	anticancer	[86]
139	(2S,3S)-pterosin C3-*O-β-D*-glucopyranoside	*Pteris multifida* Poir.		[86]
140	(2S,3S)-3-methoxypterosin	*Pteris multifida* Poir.		[86]
141	(2S,3S)-pterosin C	*Pteris multifida* Poir.		[86]
142	(2S,3S)-pterosin C	*Pteris multifida* Poir.		[86]
143	(2R)-pterosin B	*Pteris multifida* Poir.		[86]
144	(2R)-pterosin B 4-*O*-*β*-*D*-glucopyranoside	*Pteris multifida* Poir.		[86]
145	(3R)-pterosin D	*Pteris multifida* Poir.		[86]
146	(3R)-pterosin W	*Pteris multifida* Poir.		[86]
147	(3R)-pteroside W	*Pteris multifida* Poir.		[86]
148	(2R,3R)-pterosin L	*Pteris multifida* Poir.		[86]
149	Pterosinside A	*Pteris laeta* Walls	neuroprotective	[37]
150	Pterosinside B	*Pteris laeta* Walls	neuroprotective	[37]
151	Pterosinside C	*Pteris laeta* Walls	neuroprotective	[37]
152	8-Hydroxy-(-)-isolariciresinol-9-*O*-*β*-*D*-xylopyranoside.	*Pteris laeta* Walls		[37]
153	(+)-(7S, 8R, 7′S, 8′S)-8-hydroxysyringaresinol-8-*β*-*D*-xylocoside]	*Pteris laeta* Walls		[37]
154	(+)-(7S, 8R, 7′S, 8′S)-8-hydroxysyringaresinol-8-*β*-*D*-xylocoside	*Pteris laeta* Walls		[37]
155	Pteroside C	*Pteris laeta* Walls	neuroprotective	[37]
156	Pteroside B	*Pteris laeta* Walls	neuroprotective	[37]
157	Pterosin E (unidentified chirality)	*Pteris laeta* Walls	neuroprotective	[37]
158	Pterosin Z (unidentified chirality)	*Pteris laeta* Walls	neuroprotective	[37]
159	Pterosin D (unidentified chirality)	*Pteris laeta* Walls	neuroprotective	[37]
160	Pterosin X (unidentified chirality)	*Pteris laeta* Walls	neuroprotective	[37]
161	Pterosin B (unidentified chirality)	*Pteris laeta* Walls	neuroprotective 200 times Ginsenoside Rg1	[37]
162	Dehydrated pterosin S	*Pteris laeta* Walls	neuroprotective	[37]
163	Pterosin S (unidentified chirality)	*Pteris laeta* Walls	neuroprotective	[37]
164	Pterosin C (unidentified chirality)	*Pteris laeta* Walls	neuroprotective	[37]
165	2-hydroxypterosin C	*Pteris laeta* Walls	neuroprotective	[37]
166	Pterosin A (unidentified chirality)	*Pteris laeta* Walls	neuroprotective	[37]
167	Pterosin T (unidentified chirality)	*Pteris laeta* Walls	neuroprotective	[37]
168	1-hydroxy-2-epipinoresinol-1-*β-D*-glucoside	*Pteris laeta* Walls	neuroprotective	[37]
169	Schilignan F	*Pteris laeta* Walls	neuroprotective	[37]
170	1-hydroxypinoresinol-1-*β-D*-glucoside	*Pteris laeta* Walls	neuroprotective	[37]
171	Obtupterosin A	*Pteris obtusiloba* Ching & S.H. Wu	cytotoxic	[92]
172	Obtupterosin B	*Pteris obtusiloba* Ching & S.H. Wu	cytotoxic	[92]
173	Obtupterosin C	*Pteris obtusiloba* Ching & S.H. Wu	cytotoxic	[92]
174	Dehydropterosin B	*Pteris obtusiloba* Ching & S.H. Wu		[92]
175	Dehydropterosin Q	*Pteris obtusiloba* Ching & S.H. Wu		[92]
176	(2S)-pterosin P	*Pteris obtusiloba* Ching & S.H. Wu		[92]
177	(2S,3S)-pterosin S	*Pteris obtusiloba* Ching & S.H. Wu		[92]
178	(2R,3S)-pterosin S	*Pteris obtusiloba* Ching & S.H. Wu		[92]
179	(2S)-pterosin B 14-*O-β-D*-glucopyranoside	*Pteris obtusiloba* Ching & S.H. Wu		[92]
180	(3R)-pteroside W	*Pteris obtusiloba* Ching & S.H. Wu		[92]
181	Pterosin S 14-*O-β-D*-glucopyranoside	*Pteris obtusiloba* Ching & S.H. Wu		[92]
182	Creticolacton A	*Pteris cretica L.*	cytotoxic	[93]
183	13-hydroxy-2(R),3(R)-pterosin L	*Pteris cretica L.*	cytotoxic	[93]
184	Creticoside A	*Pteris cretica L.*		[93]
185	Spelosin 3-*O-β-D*-glucopyranoside	*Pteris cretica L.*		[93]
186	Pterosin D	*Pteris cretica L.*		[93]
187	(3R)-pterosin W	*Pteris cretica L.*		[93]
188	(3R)-pterosin D 3*-O-β-D*-glucopyranoside	*Pteris cretica L.*		[93]
189	(3R)-pteroside W	*Pteris cretica L.*		[93]
190	2R,3R-pterosin L 3-*O-β-D*-glucopyranoside	*Pteris cretica L.*		[93]
191	Geopyxin B	*Pteris dispar* Kunze	*cytotoxic*	[23]
192	Geopyxin E	*Pteris dispar* Kunze	*cytotoxic*	[23]
193	Ent-11*α*-hydroxy-18-acetoxykaur-16-ene	*Pteris dispar* Kunze		[23]
194	Ent-14*β*-hydroxy-18-acetoxykaur-16-ene	*Pteris dispar* Kunze		[23]
195	Neolaxiflorin L	*Pteris dispar* Kunze		[23]
196	Ent-3*β*, 19-dihydroxy-kaur-16-ene	*Pteris dispar* Kunze		[23]
197	Ent-3*β*-hydroxy-kaur-16-ene	*Pteris dispar* Kunze		[23]
198	7*β*, 17-dihydroxy-16*α*-ent-kauran-19-oic acid 19-*O*-*β*-*D*-glucopyranoside ester	*Pteris dispar* Kunze		[23]
200	Crotonkinin C	*Pteris dispar* Kunze		[23]
201	Ent-kaurane-6*β*,16*α*-diol-3-one	*Pteris ensiformis* Burm.		[97]
202	Ent-7*α*,9*α*-dihydroxy-15-oxokaur-16-en-196*β*-olide	*Pteris ensiformis* Burm.	cytotoxic	[97]
203	Ent-11*α*-hydroxy-15-oxokaur-16-en-19-oic acid	*Pteris ensiformis* Burm.	cytotoxic	[97]
204	9, 15*β*-dihydroxy-ent-kaur-16-en-19-oic acid	*Pteris ensiformis* Burm.		[97]
205	Foliol	*Pteris ensiformis* Burm.		[97]
206	(16R)-11*β*-hydroxy-15-oxo-ent-kauran-19-oic acid	*Pteris ensiformis* Burm.		[97]
207	Pterosin C (unidentified chirality)	*Pteris ensiformis* Burm.		[97]
208	Pterosin D	*Pteris ensiformis* Burm.		[97]
209	Pterosin L	*Pteris ensiformis* Burm.		[97]
210	Aspleniumside A	*Asplenium ruprechtii* Sa. Kurata	cytotoxic	[98]
211	Aspleniumside B	*Asplenium ruprechtii* Sa. Kurata	cytotoxic	[98]
212	Aspleniumside C	*Asplenium ruprechtii* Sa. Kurata	cytotoxic	[98]
213	Aspleniumside D	*Asplenium ruprechtii* Sa. Kurata		[98]
214	Aspleniumside E	*Asplenium ruprechtii* Sa. Kurata		[98]
215	Aspleniumside F	*Asplenium ruprechtii* Sa. Kurata		[100]
216	Aspleniumside G	*Asplenium ruprechtii* Sa. Kurata		[100]
217	Aspleniumside H	*Asplenium ruprechtii* Sa. Kurata		[100]
218	Aspleniumside I	*Asplenium ruprechtii* Sa. Kurata		[100]
219	3*β*,23, 24, 25, 30-pentahydroxycycloartane-3, 25, 30-tri-*O*-*β*-*D*-glucopyranoside	*Camptosorus sibiricus* Rupr.		[101]
220	3*β*,23,24,30-tetrahydroxycycloartane-25-en-3-*O*-*β*-*D*-glucopyranosyl-(1→4)-*β*-*D*-glucopyranosyl-(1→2)-*β*-*D*-glucopyranoside	*Camptosorus sibiricus* Rupr.		[101]
221	3*β*,23,24,30-tetrahydroxycycloartane-25-en-3-*O*-*β*-*D*-Glucopyranosyl-(1→2)-*β*-*D*-glucopyranosyl-(1→2)-*β*-*D*-glucopyranosyl–(1→4)-*β*-*D*-glucopyranoside	*Camptosorus sibiricus* Rupr.		[101]
222	Kaempferol-3-*O*-*β*-*D*-glucopyranoside	*Camptosorus sibiricus* Rupr.		[101]
223	Kaempferol-3, 7-*O-β-D*-diglucopyranoside	*Camptosorus sibiricus* Rupr.		[101]
224	Kaempferol-3-*O-β-D*-glucoside-7-*O*-*α*-L-rhamnoside	*Camptosorus sibiricus* Rupr.		[101]
225	Kaempferol-3-*O*-*β*-*D*-glucopyranosyl-(1→2)-*β-D*-glucopyranoside	*Camptosorus sibiricus* Rupr.		[101]
226	Camsibriside B	*Camptosorus sibiricus* Rupr.		[101]
227	Camsibriside A	*Camptosorus sibiricus* Rupr.		[101]
228	Camptoside	*Camptosorus sibiricus* Rupr.	NO inhibitor	[38]
229	Dehydrodiconiferyl alcohol-9′-*O-β-D*-glucopyranoside	*Camptosorus sibiricus* Rupr.	NO inhibitor	[38]
230	Aesculetin	*Camptosorus sibiricus* Rupr.	NO inhibitor	[38]
231	Vajicoside	*Camptosorus sibiricus* Rupr.		[38]
232	(5R,6S,8R,9S,10R)-6-*O*-[β-D-glucopyranosyl-(1→4)-*α*-l-rhamnopyranosyl] cleroda-3,13(16),14-diene	*Dicranopteris pedata* (Houtt) Nakaike	weak cytotoxicity	[102]
233	(5R,6S,8R,9S,10R,13S)-6-*O*-[β-D-glucopyranosyl-(1→4)-*α*-l-rhamnopyranosyl]-2-ox-oneocleroda-3,13-dien-15-ol.	*Dicranopteris pedata* (Houtt) Nakaike	weak cytotoxicity	[102]
234	(5R,6S,8R,9S,10R)-6-*O*-[β D-glucopyranosyl-(1→4)-*α*-l-rhamnopyranosyl]-(13E)-2-oxoneocleroda-3,14-dien-13-ol	*Dicranopteris pedata* (Houtt) Nakaike	weak cytotoxicity	[102]
235	Dryofraterpene A	*Dracaena fragrans* (L.) Schott	cytotoxic	[39]
236	Yomogin	*Dracaena fragrans* (L.) Schott		[39]
237	Pinoresinol	*Dracaena fragrans* (L.) Schott		[39]
238	(2R)-3-(4′-Hydroxyphenyl) lactic acid	*Diplazium esculentum* (Retz.) Sw.		[63]
239	Ponastrone A	*Diplazium esculentum* (Retz.) Sw.		[63]
240	Amarasterone A1	*Diplazium esculentum* (Retz.) Sw.		[63]
241	Makisterone C	*Diplazium esculentum* (Retz.) Sw.		[63]
242	Matteucens I	*Matteuccia orientalis* (Hook.) Trevis.		[40]
243	Matteucens J	*Matteuccia orientalis* (Hook.) Trevis.		[40]
244	Demethylmatteucinol	*Matteuccia intermedia* C.Chr.		[44]
245	Matteflavosides H	*Matteuccia intermedia* C.Chr.		[44]
246	Matteflavosides I	*Matteuccia intermedia* C.Chr.		[44]
247	Matteflavoside J	*Matteuccia intermedia* C.Chr.		[44]
248	Matteuinterate A	*Matteuccia intermedia* C.Chr.		[44]
249	Matteuinterate B	*Matteuccia intermedia* C.Chr.		[44]
250	Matteuinterate C	*Matteuccia intermedia* C.Chr.		[44]
251	Matteuinterate D	*Matteuccia intermedia* C.Chr.		[44]
252	Matteuinterate E	*Matteuccia intermedia* C.Chr.		[44]
253	Matteuinterate F	*Matteuccia intermedia* C.Chr.		[44]
254	Demethoxymatteucinol	*Matteuccia intermedia* C.Chr.		[44]
255	Farrerol	*Matteuccia intermedia* C.Chr.	*α*-Glucosidase inhibitory	[44]
256	Matteucin	*Matteuccia intermedia* C.Chr.	*α*-Glucosidase inhibitory	[44]
257	Matteucinol	*Matteuccia intermedia* C.Chr.	*α*-Glucosidase inhibitory	[44]
258	Methoxymatteucin	*Matteuccia intermedia* C.Chr.	*α*-Glucosidase inhibitory	[44]
259	3′-hydroxy-matteucinol	*Matteuccia intermedia* C.Chr.	*α*-Glucosidase inhibitory	[44]
260	Cyrtominetin	*Matteuccia intermedia* C.Chr.	*α*-Glucosidase inhibitory	[44]
261	5,7-dihydroxy-4′-methoxy-6-methyl-flavanone	*Matteuccia intermedia* C.Chr.		[44]
262	Demethoxymateucinol 7*-O*-glucoside	*Matteuccia intermedia* C.Chr.		[44]
263	Farrerol 7-*O*-glucoside	*Matteuccia intermedia* C.Chr.		[44]
264	Matteucinol 7-*O*-glucoside	*Matteuccia intermedia* C.Chr.		[44]
265	Myrciacitrin II	*Matteuccia intermedia* C.Chr.		[44]
266	Matteflavoside G	*Matteuccia intermedia* C.Chr.		[44]
267	Matteuorienate A	*Matteuccia intermedia* C.Chr.		[44]
268	Matteuorienate B	*Matteuccia intermedia* C.Chr.		[44]
269	Matteuorienate D	*Matteuccia intermedia* C.Chr.		[44]
270	Matteuorienate F	*Matteuccia intermedia* C.Chr.		[44]
271	Matteuorienate H	*Matteuccia intermedia* C.Chr.		[44]
272	Matteuorienate I	*Matteuccia intermedia* C.Chr.		[44]
273	Matteuorienate J	*Matteuccia intermedia* C.Chr.		[44]
274	Matteuorienate K	*Matteuccia intermedia* C.Chr.		[44]
275	Matteuinterin A	*Matteuccia intermedia* C.Chr.		[39]
276	Matteuinterin B	*Matteuccia intermedia* C.Chr.		[39]
277	Matteuinterin C	*Matteuccia intermedia* C.Chr.		[39]
278	Kankanoside P	*Matteuccia intermedia* C.Chr.		[39]
279	(3S,6S)-6,7-dihydroxy-6,7-dihydrolinalool 3-*O-β-D*-glucopyranoside	*Matteuccia intermedia* C.Chr.	anti-inflammatory	[39]
280	(6R,7E,9R)-9-hydroxy-4,7-megastigmadien-3-one 9-*O-β-D*-glucopyranoside	*Matteuccia intermedia* C.Chr.		[39]
281	(6S,7E,9R)-9-hydroxy-4,7-megastigmadien-3-one 9-*O-β-D*-gluco-pyranoside	*Matteuccia intermedia* C.Chr.		[39]
282	Byzantionoside B	*Matteuccia intermedia* C.Chr.		[39]
283	Isodonmegastigmane I	*Matteuccia intermedia* C.Chr.		[39]
284	9-*O*-*β*-*D*-glucopyranosyloxy-5-megastigmen-4-one	*Matteuccia intermedia* C.Chr.	anti-inflammatory	[39]
285	Eriodictyol-7-*O*-glucuronide	*Tectaria coadunata* (Wall. ex Hook. & Grev.) C.Chr		[47]
286	Luteolin-7-*O*-glucoronide	*Tectaria coadunata* (Wall. ex Hook. & Grev.) C.Chr		[47]
287	Dryocrasspherol A	*Dryopteris crassirhizoma* Nakai	anti-respiratory syncytial virus activity	[26]
288	Dryocrasspherol B	*Dryopteris crassirhizoma* Nakai		[26]
289	Dryocrasspherol C	*Dryopteris crassirhizoma* Nakai		[26]
290	Dryocrasspherol D	*Dryopteris crassirhizoma* Nakai	anti-inflammatory	[26]
291	Dryocrasspherol E	*Dryopteris crassirhizoma* Nakai	anti-inflammatory	[26]
292	Fragranoside B	*Dracaena fragrans* (L.) Schott		[48]
293	Dihydroconiferyl alcohol	*Dracaena fragrans* (L.) Schott		[48]
294	1,3-dihydroxy-5-propylbenzene	*Dracaena fragrans* (L.) Schott	*anticancer*	[48]
295	4-hydroxyacetophenone	*Dracaena fragrans* (L.) Schott		[48]
296	3,4-dihydroxybenzaldehyde	*Dracaena fragrans* (L.) Schott		[48]
297	2-ethyl-6-hydroxybenzoic acid	*Dracaena fragrans* (L.) Schott		[48]
298	3,4-dihydroxyacetophenone	*Dracaena fragrans* (L.) Schott	*anticancer*	[48]
299	(+)-afzelechin-7-*O*-*α*-*L*-arabinofuranoside	*Polypodium vulgare* L.		[1]
300	(+)-afzelechin-7-*O-β-D*-apiofuranoside	*Polypodium vulgare* L.		[1]
301	(+)-afzelechin	*Polypodium vulgare* L.		[1]
302	(+)-catechin-7-*O*-*α*-*L*-arabinofuranoside	*Polypodium vulgare* L.		[1]
303	(+)-catechin-7-*O-β-D*-apiofuranoside	*Polypodium vulgare* L.		[1]
304	Liglaurate A	*Drynaria roosii* Nakaike	*a* *ntioxidant, anti-inflammatory, and cytotoxic*	[52]
305	Liglaurate B	*Drynaria roosii* Nakaike	*a* *ntioxidant, anti-inflammatory, and cytotoxic*	[52]
306	Liglaurate C	*Drynaria roosii* Nakaike	*a* *ntioxidant, anti-inflammatory, and cytotoxic*	[52]
307	Liglaurate D	*Drynaria roosii* Nakaike	*a* *ntioxidant, anti-inflammatory, and cytotoxic*	[52]
308	Liglaurate E	*Drynaria roosii* Nakaike		[52]
309	Methyl 12-caffeoyloxylaurate	*Drynaria roosii* Nakaike		[52]
310	Glyceryl 12-caffeoyloxylaurate	*Drynaria roosii* Nakaike	*a* *ntioxidant, anti-inflammatory*	[52]
311	Protocatechuic acid methyl ester-4-*O*-(6′-*O*-proto-catEchuoyl)-*β*-*D*-glucopyranoside	*Phymatopteris hastata* (Thunb.) Pic. Serm.	*α-glucosidase inhibitory*	[53]
312	(-)/(+)-naringenin-7-*O*-*α*-*L*-rhamnopyranoside	*Phymatopteris hastata* (Thunb.) Pic. Serm.		[53]
313	Coumarin	*Phymatopteris hastata* (Thunb.) Pic. Serm.	*α-glucosidase inhibitory*	[53]
314	Trans-*O*-coumaric acid	*Phymatopteris hastata* (Thunb.) Pic. Serm.		[53]
315	Trans-caffeic acid	*Phymatopteris hastata* (Thunb.) Pic. Serm.		[53]
316	Juglanin	*Phymatopteris hastata* (Thunb.) Pic. Serm.		[53]
317	Kaempferol 3-*O*-*α*-*L*-rhamnopyranoside	*Phymatopteris hastata* (Thunb.) Pic. Serm.		[53]
318	Kaempferol-7-*O*-*α*-*L*-rhamnopyranoside	*Phymatopteris hastata* (Thunb.) Pic. Serm.		[53]
319	Avicularin	*Phymatopteris hastata* (Thunb.) Pic. Serm.		[53]
320	Zeaxanthin	*Phymatopteris hastata* (Thunb.) Pic. Serm.	*α-glucosidase inhibitory*	[53]
321	Kaempferol-3-O*-α-L*-arabinofuranosyl-7-*O-α-L*-rhamnopyranoside	*Phymatopteris hastata* (Thunb.) Pic. Serm.	*α-glucosidase inhibitory*	[53]
322	Kaempferol 3-*O*-[*α*-*D*-apiofuranosyl-(1–2)-*α-L*-arabinofuranoside]-7-*O*-*α*-*L*-rhamnopyranoside	*Phymatopteris hastata* (Thunb.) Pic. Serm.		[53]
323	β-ecdysterone	*Phymatopteris hastata* (Thunb.) Pic. Serm.		[53]
324	Trans-melilotoside	*Phymatopteris hastata* (Thunb.) Pic. Serm.		[53]
325	(-)-epi-osmundalactone	*Angiopteris helferiana* C.Presl		[54]
326	Angiopteroside	*Angiopteris helferiana* C.Presl		[54]
327	2-deprenyl-5-*O*-methyl-7-methoxy-rheediaxanthone B	*Metaxya rostrata* (Kunth) C.Presl	anticancer	[60]
328	2-deprenyl-6-*O*-methyl-7-hydroxy-rheediaxanthone B	*Metaxya rostrata* (Kunth) C.Presl	anticancer	[60]
329	2-deprenyl-5-*O*-methyl-7-hydroxy-rheediaxanthone B	*Metaxya rostrata* (Kunth) C.Presl	anticancer	[60]
330	4-*O*-coumaroyl-*β-D*-allose	*Cibotium barometz* (L.) J. Sm.	hepatoprotective, anti-inflammatory	[42]
331	4-*O*-caffeoyl-*β-D*-allose	*Cibotium barometz* (L.) J. Sm.		[42]
332	Ethyl (4-O-caffeoyl)*-β-D*-glucopyranoside	*Cibotium barometz* (L.) J. Sm.		[42]
333	Cibotiumbaroside D	*Cibotium barometz* (L.) J. Sm.	hepatoprotective	[42]
334	4-*O*-coumaroylglucose	*Cibotium barometz* (L.) J. Sm.		[42]
335	6-*O*-coumaroylglucose	*Cibotium barometz* (L.) J. Sm.		[42]
336	4-*O*-caffeoylglucose	*Cibotium barometz* (L.) J. Sm.	hepatoprotective	[42]
337	6-*O*-caffeoylglucose	*Cibotium barometz* (L.) J. Sm.		[42]
338	Caffeic acid 4-*O-β-D*-glucopyranoside	*Cibotium barometz* (L.) J. Sm.	anti-inflammatory	[42]
339	Ferulic acid 4-*O-β-D*-glucopyranoside	*Cibotium barometz* (L.) J. Sm.	hepatoprotective	[42]
340	Vanillic acid 4-*O-β-D*-glucopyranoside	*Cibotium barometz* (L.) J. Sm.		[42]
341	Cyathenosin A	*Cibotium barometz* (L.) J. Sm.		[42]
342	Jixueqisus A	*Pronephrium penangianum* (Hook.) Holtt		[61]
343	Jixueqisus B	*Pronephrium penangianum* (Hook.) Holtt		[61]
344	Jixueqisus C	*Pronephrium penangianum* (Hook.) Holtt		[61]
345	Jixueqisus D	*Pronephrium penangianum* (Hook.) Holtt		[61]
346	Jixueqisus E	*Pronephrium penangianum* (Hook.) Holtt		[61]
347	Jixueqisus F	*Pronephrium penangianum* (Hook.) Holtt		[61]
348	(2S)-5,2′,5′-trihydroxy-7-methoxyflavanone	*Pronephrium penangianum* (Hook.) Holtt		[61]
349	5,2′, 5′-trihydroxy-7-methoxyflavone	*Pronephrium penangianum* (Hook.) Holtt		[61]
350	Abacopterin C	*Pronephrium penangianum* (Hook.) Holtt	cytotoxic	[61]
351	Abacopterin A	*Pronephrium penangianum* (Hook.) Holtt	cytotoxic	[61]
352	Eruberin B	*Pronephrium penangianum* (Hook.) Holtt	cytotoxic	[61]
353	Triphyllin A	*Pronephrium penangianum* (Hook.) Holtt	cytotoxic	[61]
354	Eruberin A	*Pronephrium penangianum* (Hook.) Holtt		[61]
355	Abacopterin I	*Pronephrium penangianum* (Hook.) Holtt		[61]
356	Abacopterin K	*Pronephrium penangianum* (Hook.) Holtt		[61]
357	(S, E)-7-(2-octylcyclopropylidene) heptanoic acid (Adiantic acid)	*Adiantum flabellulatum* L.		[67]
358	Stigmast-4-en-6-β-ol-3-one	*Adiantum flabellulatum* L.		[67]
359	β-sitosterol	*Adiantum flabellulatum* L.		[67]
360	Isoadiantol A	*Adiantum flabellulatum* L.		[67]

#### 3.4.3. Gleicheniaceae Family

Despite its widespread distribution in marine organisms and the plant kingdom, clerodane diterpenes were reported only in ferns from five species of the genus *Dicranopteris*, which varied according to the junction position between the sugar and the decalin ring or the C-9 moiety [102]. *Dicranopteris pedata* (Houtt.) Nakaike introduced three new clerodane molecules **333-335** (Figure 9) with weak cytotoxic activity. All the structures were of high resemblance to the known compound (6S,13S)-6-{[*β*-*D*-glucopyranosyl-(1-4)-*α*-*L*-rhamnopyranosyl] oxy} cleroda 3,14-dien-13-ol except in the attachment of the sugars and the position of the hydroxyl or methyl groups in one or two positions [103].

#### 3.4.4. Dryopteridaceae Family

From *Dracaena fragrans* (L.) Schott, the new sesquiterpene dryofraterpene A **336** (Figure 9) or the (7S,10S)-2,3-dihydroxy-calamenene-15-carboxylic acid methyl ester was isolated and characterized. Its antitumor activity was revealed against the cell lines A549, MCF7, HepG2, HeLa, and PC-3 with an IC_50_ below 10 μM compared to taxol as a positive control [104].

#### 3.4.5. Athyriaceae Family

Gymnomitrane sesquiterpenes were newly reported from ferns and included matteuinterin A **339** (Figure 9). Interestingly, **340** showed a potent anti-inflammatory effect by reducing PGE2 in lipopolysaccharide (LPS)-induced RAW 264.7 murine macrophages with an IC_50_ value of 17 μM, which was even better than the minocycline control [44]. Gymnomitrane sesquiterpenes were detected from plants, liverworts, and fungi, namely, *Codonopsis pilosula* (Franch.) *Nannf.*, *Ganoderma lucida* (Lingzhi or Reishi) [105], basidiomycete *Antrodiella albocinnamomea* [106], *Cylindrocolea recurvifolia* [Steph.] Inoue [107], and *Jungermannia truncata* NEES [108]. While matteuinterin A was identified by analogy of its aglycone part to 3-gymnomitren-15-ol, matteuinterin B displayed a resemblance to leptorumol 7-*O*-*β*-*D*-glucopyranoside, yet the attachment of the 3-hydroxy-3-methylglutaryl (HMG) fragment to the C-6′ position was not seen before and was confirmed by HMBC [44].

### 3.5. Polysaccharides

The widespread use of calorie- and simple sugar-rich foods, which are usually devoid of any dietary fibers, vitamins, or complex carbohydrates, throughout the previous decades was implicated in the obesity epidemic and its chronic consequences such as stroke, cardiovascular diseases, and cancer. Accordingly, scientific attention was drawn towards using functional foods and their phytochemical components for their undeniable various health-promoting aspects. Among other components are polysaccharides from different sources such as algae and plants, which are reported to have prebiotic, anticancer, antidiabetic, antiviral, immunomodulatory, wound-healing, and antibacterial effects [109]. Probably the most promising are the water-soluble polysaccharides based on their safety profile and variable bioactivities, for instance, antihyperglycemic, immunomodulatory, and antitumor activities [110,111,112]. The complex carbohydrate characterization depends on the chemical composition of monomers, the position of branches, molecular weight, and the type of glycosidic links [66].

BTP1 polysaccharide was obtained from *Botrychium ternatum* (Thunb.) Sw.; family *Ophiolossaceaus*. This water-soluble polysaccharide with a molecular weight of 11,638 Da according to the MALDI matrix was predicted to be a good immunomodulatory agent that could elevate the medicinal and nutritious value of the traditional Chinese fern *Botrychium ternatum* (Thunb.) Sw. Its chemical structure was determined putatively both by MALDI-TOF and NMR to be (1→)-linked *α*-*L*-Araf at *O*-2, *O*-3, and adjacent *O*-2 positions lying over a backbone of linear (1→5)-Araf. In the RAW 254.7 cell assays, BTP1 proved to enhance cell viability and promote NO release [66].

The polysaccharide DCP-3 was isolated from the fern *Dryopteris crassirhizoma* Nakai, which is used as a traditional antiviral agent in China. DCP-3 is composed of monomer units of mannose (9.22%), galactose (36.65%), arabinose (17.07%), and xylose (34.75%) arranged in an unprecedented triple helical structure with a molecular weight of 273.2 kDa. The ferric reducing power, hydroxyl radical, and DPPH scavenging radical activity were measured and revealed the potency of DCP-3 as an antioxidant; moreover, DCP-3 stimulated RAW264.7 macrophages and enhanced NO production. These results were encouraging enough to support the plausible use of *Dryopteris crassirhizoma* Nakai fern in the food industry and nutraceuticals [113].

*Athyrium multidentatum* Ching (AMC) polysaccharides were investigated by Ching et al., who revealed six main monomers, namely, arabinose, galactose, glucose, glucuronic acid, rhamnose, mannose, and fucose, with a total molecular weight of 33,203 Da isolated from the rhizomes. The chemical characterization was performed by NMR, FT-IR, monosaccharide analysis, and molecular weight determination; furthermore, the compounds’ antiaging properties were studied in the mouse model of *D*-galactose-induced aging. The results showed a significant reversal of the changes induced by *d*-galactose, particularly an elevated Bcl-2/Bax ratio, lowered caspase-3 level, Akt phosphorylation, enhanced Akt mRNA expression levels, and heme oxygenase-1 (HO-1) together with nuclear factor-erythroid 2-related factor 2 (Nrf2) mRNA expression. These outcomes supported the hypothesis that AMC polysaccharides could be implicated in antiaging or antiwrinkle preparations by affecting the PI3K/Akt/Nrf2 and FOXO3a pathways and their downstream antioxidant factors [114].

## 4. Health-Promoting Effects of Ferns

### 4.1. Anti-Inflammatory and Antioxidant Effects

Four fern extracts from Osmunda japonica, *Matteuccia* species *struthiopteris* and *orientalis*, and *Pteris aquilinum* were studied for their antioxidant and anti-inflammatory activities in two inflammatory pathways: gene expression inhibition of IL6 and IL1β, and iNOS gene expression by LPS-induced macrophages. All fern extracts reduced the IL1β gene expression, especially the *Osmunda japonica* roots and *Malus orientalis* (Hook.) Trev. Fronds, where their IC_50_ values were reported to be 17.8 and 50.0 µg/mL, respectively. The extract of *Malus orientalis* (Hook.) Trev fronds demonstrated a reduction in the iNOS pathway at a relatively low concentration of 20 µg/mL [115]. In vitro assays conducted using the *Athyrium multidentatum* (Doll.) Ching (AM) aerial parts in the lipopolysaccharide (LPS)-induced inflammatory model indicated that the AM extract lowered the gene expression of the two synthetases (iNOS and COX-2), leading to the downregulation of NO and PGE2 [116] (Table S1).

*Stenochlaena palustris* J. Sm. was highlighted as a favorable vegetable functional food that is rich year-round in polyphenols, anthocyanins, and flavonoids; moreover, antioxidant assays such as the ferric reducing and radical scavenging activities revealed that the total polyphenolic content of *S. palustris* extracts was 51.69 mg/g [117]. *Dryopteris erythrosora* (D.C. Eaton) Kuntze was one of the fern plants whose total flavonoid content was reciprocal to its antioxidant power as compared to their rutin equivalents, even though the ABTS, DPPH, superoxide anion scavenging, and FRAP were comparable to rutin. This was possibly because of the cytotoxic action of the flavonoids gliricidin 7-*O*-hexoside, quercetin 7-*O*-rutinoside, quercetin 7-*O*-galactoside, apigenin 7-*O*-glucoside keampferol 7-*O*-gentiobioside, myricetin 3-*O*-rhamnoside, and keampferol-3-*O*-rutinoside. The *Dryopteris erythrosora* (D.C. Eaton) Kuntze flavonoids exerted an acetylcholinesterase effect leading to their anti-inflammatory and/or antioxidant properties [118]. Among the highly nutritious ferns were the three potherbs *Osmunda cinnamomea Linn*, *Pteridium aquilinum* (L.) Kuhn, and *Athyrium multidentatum* (Doll.). A strong antioxidant power was attributed to them, especially *Athyrium multidentatum* (Doll.). [119]*. Diplazium maximum* [D. Don] displayed richness in phenolics, with procatechuic acid, myricetin, epicatechin, and catechin constituting noteworthy quantities, which bestows *Diplazium maximum* with the potential to be consumed widely as an instant functional food [120].

*Asparagus officinalis* L. Spears was rich in phenolics, giving it a superior antioxidant potential over other vegetables. Upon studying the various antioxidant assays such as the cupric reducing antioxidant capacity (CUPRAC) and ferric reducing/antioxidant power (FRAP), the extracts of Asparagus showed different contents of bioactive compounds, as the phenolics reached up to 16.9 mg GAE/g. Asparagus was favored as an important dietary component [121] (Table S1). Similarly, the *Stenoloma chusanum* (L.) Ching subterranean parts total flavonoid content (TFC) and total phenolic content (TPC) were quantified to be 24.63 ± 1.34% and 9.58 ± 0.41%, respectively [122]. In a recent study, more than 30 edible ferns from 13 families grown in Europe were examined for their antioxidant activity and nutraceutical application. The genera were noncytotoxic against ovine hepatocytes and included the following: *Asplenium*, *Athyrium*, *Blechnum*, *Davallia*, *Pteridium*, *Dryopteris*, *Polystichum*, *Marsilea*, *Regnellidium*, *Matteuccia*, *Osmunda*, *Polypodium*, *Adiantum*, *Lastrea*, *Phegopteris*, *Thelypteris*, and *Cystopteris* [123]. Compared to rocket and spinach vegetables, most of the species were revealed as good sources of carotenoids, for example, lutein (205 µg·g^−1^ dry weight) and β-carotene (161 µg·g^−1^ dry weight). The high antioxidant effect of these ferns exceeded 0.5 g Trolox eq/gm dry weight in the ORAC assay, with an IC_50_ value of less than 30 µg·mL^−1^ in the DPPH assays [124].

*Isoetes sinensis* Palmer is an endangered fern with a relatively high total flavonoid content of 10.74 ± 0.25 mg/g. Compounds such as flavones; apigenin-7-glucuronide, acacetin-7-*O*-glcopyranoside, and homoplantageninisoetin; one prodelphinidin (procyanidins) and one nothofagin (dihydrochalcone); and four flavonols, namely, isoetin, kaempferol-3-*O*-glucoside, quercetin-3-*O*-[6″-*O*-(3-hydroxy-3-methyl glutaryl)-*β*-*D*-glucopyranoside], and limocitrin-Neo, were detected in the mass spectrometry-DAD at 254 nm [125] (Table S1). Traditionally, ferns such as Diplazium and Pteridium were included as pickles in the Himalayans’ diet [126].

Even though *Dryopteris crassirhizoma* (DC) was used in folk medicine as anti-inflammatory and antibacterial agent, in vivo studies manifested its effectiveness in the treatment of allergic rhinitis through the suppression of Th2 cytokine overproduction and mast cell infiltration and the reduction of nasal fluid Treg cytokines [127]. *Polypodium vulgare* L. (Polypodiaceae) showed an antioxidant effect, possibly due to the high polyphenolic content, cellular repair activity in 3T3 fibroblast cells, as well as cytoprotective potential with no appreciable cytotoxicity in physiological conditions [128]. *Adiantum capillus-veneris* Linn. (ACVL) revealed a protective effect against inflammatory reactions induced by carbendazim pesticide (CBZ) in Sprague–Dawley female rats. Reduced CPZ toxicity and symptom reversal were recorded after the administration of the fern due to its anti-inflammatory effect and inhibition of NF-κB-P65 synthesis as the major inflammatory marker [129]. Ferns such as *Adiantum*, *Dryopteris*, *Blechnum*, *Pteris*, and *Azolla* species were reported as rich sources of 3-deoxyanthocyanidins, rare anthocyanin-type compounds with unique antioxidant, anticancer, and favorable food coloration effects [130]. *Pteris ensiformis* Burm. extracts were proposed as a food supplement due to their bioactivities and higher antioxidant effect in the superoxide anion and hydroxyl radical assays [131].

From the Sumatrian fern *Trichomanes javanicum Blume* (Hymenophyllaceae), mangifern revealed an activity twice as high as that of the standard ascorbic acid with an IC_50_ value of 3.89 μM. Another sumatrian fern, *Oleandra pistillaris* (Sw.) C. Chr. (Oleandraceae), is rich in gallic acid and phenolic acids and was significantly active against *Salmonella thypimurium* [132]. The leaves of *Adiantum capillus-veneris* Linn. were extracted, and thirteen phenolics were characterized putatively by reversed phase HPLC-DAD profiling; the phenolics included caftaric acid, 5-caffeoylquinic acid, hydroxybenzoic acid, chlorogenic acid, rosmarinic acid, kaempferol glycosides, *p*-coumaric, and quercetin glycosides [133]. (-)-epi-Osmundalactone and angiopteroside were studied using RAW cells, and anti-inflammatory bioactivity was revealed, especially for (-)-epi-osmundalactone [54].

### 4.2. Anti-Adipogenic Effect

The WHO has declared obesity an epidemic disease worldwide associated with many metabolic disorder diseases. Not only is obesity linked to cardiovascular aliments and hypertension but also to chronic diseases such as osteoarthritis, cancers, inflammatory pathologies, stroke, and sleep apnea. A large number and size of adipocytes is considered the main manifestation of obesity; accordingly, research interest is evolving in this area to target factors affecting adipogenic cells and inhibitors of lipid and carbohydrate digestion or absorption [54] (Table S1). Fern functional foods are among the promising ingredients that could be consumed regularly, thus promoting human health through its high-nutrient and low-fat content. The combined type 2 diabetes and obesity syndrome, denoted as diabesity, has a high occurrence rate at present. 3T3-L1 cells were used as an in vitro model of cells to assess the lipid production rate and the anti-adipogenic effect where both (-)-epi-osmundalactone and angiopteroside showed significant results with 30% and 20% inhibition rates, respectively. As far as the obesity metabolic pattern is concerned, inflammation is regarded as a key player affecting TNF-α and various immune cells to overproduce or stimulate the proliferation of adipogenic cells; therefore, it makes sense to consider the anti-inflammatory activity for promising antiadipogenic molecules. A structure correlation was deduced that linked the biological activity of both lactones with the glycosidic linkage. The absence of the glucose linkage was hypothesized to reduce the biological effectiveness as manifested by angiopteroside, which was weaker than (-)-epi-osmundalactone. Likewise, previous studies on natural flavonoids supported the hypothesis that aglycones and glycosides do have different bioavailabilities and activities [134].

*Adiantum capillus-veneris* Linn. was studied for its influence on the triglyceride profile of diabesity patients and showed in vitro efficiency against *α*-amylase/*α*-glucosidase comparable to acarbose, the standard antidiabetic drug. Surprisingly, *Adiantum capillus-veneris* Linn. was not able to hinder glucose diffusion, but it inhibited the pancreatic lipase (PL) enzyme together with its pure components ferulic, ellagic, and chlorogenic acids [135] (Table S1). The methanolic and aqueous extracts of *Tectaria coadunata* were investigated for their activity against five metabolic and degenerative *α*-glucosidase and *α*-amylase enzymes and showed promising activity. The reason was based on the rich procyanidin-type A content in the fern’s extract, mainly luteolin-7-*O*-glucoronide and eriodictyol-7-*O*-glucuronide [47]. The medicinal fern *Stenochlaena palustris* (Burm. f.) was reported as an inhibitor of *α*-glucosidase and amylase enzymes, contributing to carbohydrate digestion. These activities were shown by different fractions, particularly the polar fractions [136].

The butanol, water, and methanolic extracts of the *Angiopteris helferiana* C. Presl ferns’ rhizome were investigated in the DPPH assay to show their significant antioxidant and *α*-glucosidase activities; moreover, in 3T3-L1 cells, the three fractions revealed their promising antiadipogenic potential by inhibiting adipogenic markers (PPARγ, C/EBPα) [54].

### 4.3. Anticancer Effect

The use of nutraceuticals either to treat or protect from carcinogenic agents has been on the rise, in particular, due to their natural herbal origin, which patients or users deliberately trust as natural. Many phytochemicals such as flavonoids, carotenoids, or stilbenes have been proven to significantly enhance apoptosis through various mechanisms; thus, they are encouraged to be used in combination therapies [137,138]. Geopyxins B and E as well as other ent-kaurene derivatives manifested a moderate cytotoxic effect against the Bel-7402 and HepG2 cell lines with IC_50_ values between 6.6 and 10.6 μmol/L [95]. The water extract of *Camptosorus sibiricus* Rupr. was found to suppress ROS by activating HO-1 and NQO-1 Nrf2-mediated reductases. Furthermore, DNA damage caused by induced mutations and oxidative stress was attenuated in BEAS.2B cells and B(a)P-induced lung adenocarcinoma cells. Tumor size, volume and growth were diminished by *C. sibiricus* in *in-vivo* experiments [101]. Nine cell lines were targeted including MG63, HepG2, MB231, U251, A549, HeLa, HOS, U2OS, and SKBR-3, and the effectiveness ranged from IC_50_ 1.24 µg/mL for pinoresinol in Hela cells to 48.3 µg/mL for yomogin in HepG2 cells, representing the highest and lowest potencies, respectively [104].

Beyond the antitumor effect of seed plants and their essential oils [139], fern herbs were remarkable in their anticancer effect. Among the highly nutritious ferns are three potherbs, *Osmunda cinnamomea* Linn, *Pteridium aquilinum*, *Filicium decipiens*, and *Athyrium multidentatum* (Doll.). A significant anticancer power was attributed to the three ferns, especially *Athyrium multidentatum* (Doll.) as a biomolecule protectant and as a cellular antioxidant. On the molecular level, extracts of *Athyrium multidentatum* (Doll.) led to inhibition of wound-healing and mitochondrial membrane potential (MMP) reduction in HepG2 cells [119].

### 4.4. Neuroprotective Effect

**Figure 10:**
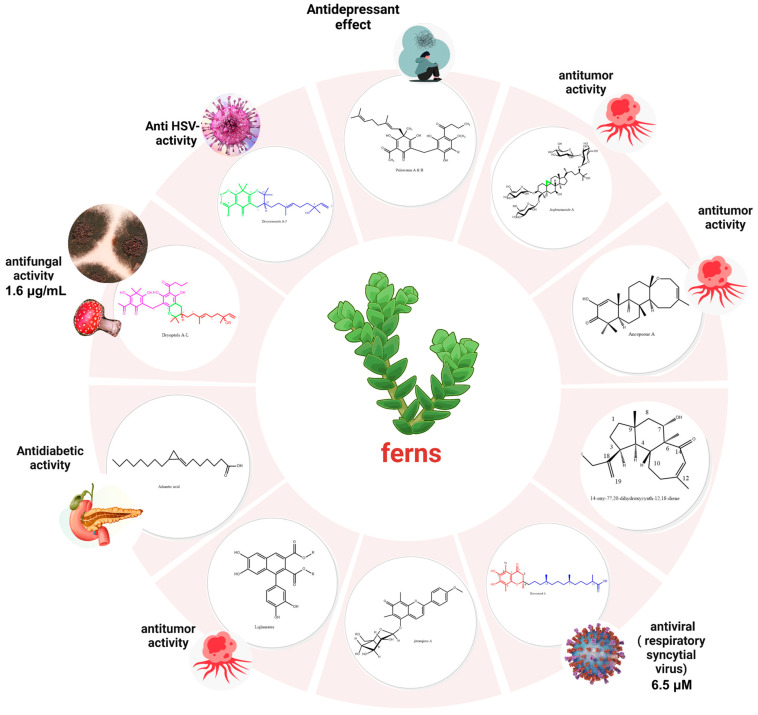
Illustration of ferns’ chemical diversity and biological effects.

The edible fern *Diplazium esculentum* [Retz.] was studied both in vitro and in vivo to examine its effects as an acetylcholine esterase inhibitor as well as an anti-amyloid B (Aβ). The Drosophila models of *α*-amyloid toxicity were affected positively by the ethanolic extracts of *Diplazium esculentum* (Retz.), which exhibited high phenolic and flavonoid contents and reduced the BACE-1 and amyloid beta 42 (Aβ42) peptides, thus ameliorating the locomotor functioning in Alzheimer disease and other dementia aliments [140] (Figure 10). The methanolic and aqueous extracts of *Tectaria coadunata* (Wall. ex-Hook. & Grev.) C. Chr. were investigated for their activity against the AChE and BChE enzymes and showed promising activity. The reason was based on the rich procyanidin-type A content in the fern’s extract, mainly luteolin-7-*O*-glucoronide and eriodictyol-7-*O*-glucuronide [47]. The structural features of flavonoids such as the number and position of OH groups and the oxidation of the C-ring were associated with anti-Alzheimer activity, particularly the phenyl chroman backbone. Catechin and epicatechin glucuronide metabolites presented the highest concentration in the blood after the consumption of polyphenol-rich food. It is worth stating that these metabolites are associated with better synaptic functioning and transmission.

Polyphenols were not only implicated in preventing AD causative factors but also type 2 diabetes since both diseases share the common Aβ aggregation in the brain and human islets of the pancreas, respectively. The antioxidant power of polyphenols contributed effectively to their beneficial roles here. While the amyloid fiber topological structures would be impeded by the aromaticity and the hydrophobic groups encountered in many phenolic compounds, AB fibrils are destabilized in their aggregation by the antioxidant effect [141]. Catechols possessed disaggregation and anti-aggregation properties, and their SARs referred to hydrogen bonding formation with Glu22 as well as the OH group. For instance, hispidin derivatives, caffeoylquinic acid, schizotenuin A, kukoamines A and B, lycopic acids, clovamide, and rosmarinic acid successfully inhibited amyloid protein aggregation [142]. The number of catechol groups was proportional to the anti-amyloid activity, as it oxidized to *O*-benzoquinone covalently bound to the *β*-sheet structure of the amyloid proteins.

*Diplazium esculentum* (Retz.) ethanolic extracts manifested promising results when tested both in vitro and in Aβ-mediated toxicity Drosophila models. A high content of phenolics and flavonoids was shown to possibly contribute to the anti-AD and antioxidant effects. The locomotor activities were improved, with a notable inhibition of the BACE-1 and amyloid beta 42 (Aβ42) peptide decline [139]. By far, Pteris sesquiterpenes showed antioxidant potential, which gives them the potential to be efficient molecules for reducing the progression of and prohibiting AD symptoms [37].

### 4.5. Skin-Protective Effect

*Asplenium australasicum* (J. Sm.) Hook, the edible bird’s nest fern, revealed effectiveness as a melanogenesis inhibitor in skin tissues based on its phytoconstituents; it is mainly rich in polyphenols, fucose-rich polysaccharides, and p-coumaric acid. The study highlighted the effect of these natural products on higher collagen production, fibroblast growth, and skin elasticity [142] (Table S1). *Polypodium leucotomos* fern (PLF) was one of the components of the Over-the-Counter (OTC) dietary dermatological preparations for skin, hair, and nails. As a dietary supplement prescribed by 66% of dermatologists, the preparation is used by a large population in the USA. Other components were biotin, vitamin D, zinc, and nicotinamide [143]. Despite the insufficient clinical trials, PLF was reported to have in vitro antioxidant and photoprotective effects that reduce UV-induced skin erythema. Various evidence supports the use of PLF in actinic keratosis and idiopathic photodermatoses [143]. On the other hand, flavonoids with a 3-hydroxy, 4-keto group were effective tyrosinase inhibitors, which is possibly due to the analogy with the di-hydroxyphenyl group in l-DOPA, and the *Tectaria coadunata* (Wall. ex-Hook. & Grev.) C. Chr. alcoholic extracts depicted a promising tyrosinase inhibitory activity that reached around 149.41 mg kojic acid equivalent (KAE)/g [47]. The methanolic and aqueous extracts of *Tectaria coadunata* (Wall. ex-Hook. & Grev.) C. Chr. were investigated for their activities against tyrosinases and showed promising activity based on the rich procyanidin-type A content in the fern’s extract, mainly luteolin-7-*O*-glucoronide and eriodictyol-7-*O*-glucuronide [47]. The dimeric phloroglucinols from *Dryopteris crassirhizoma* Nakai roots revealed their suppressing effect against melanogenesis in B16F10 murine melanoma cells with IC_50_ values between 181.3 and 35.7 μM. Among the important targets to block the melanogenesis pathway is the tyrosinase enzyme. As a tyrosinase inhibitor, norflavaspidic acid PB manifested potencies with an IC_50_ value of 5.9 μM in B16F10 cells [21], and the study recorded the vital role of the ring substituents and their functional groups in determining the degree of tyrosinase inhibition.

### 4.6. Bone Mineralization Effect

The *Davallia formosana Hayata* (DFH) water and ethanolic extracts showed effectiveness in MC3T3E1 osteoblasts. The water extract enhanced cell survival and increased the bone morphogenetic and maturation markers BMP-2, RUNX-2, ALP, and CoL-1 and, hence, cell differentiation. Moreover, late mineralization was stimulated by both extracts. Upon studying the metabolic constituents of the water extract of DFH, (-)-epicatechin-3-*O*-*D*-allipyranoside was isolated and revealed superior results in promoting mineralization (218.7%) and cell survival (118.9%) [123] (Table S1).

### 4.7. Immunomodulatory Effect

*Pteridium aquilinum* (L.) Kuhn was the source of the PAP1-A polysaccharide composed of the monomers *L*-fucose, *D*-galactose, *L*-rhamnose, *D*-xylose, *D*-mannose, *L*-arabinose, and *D*-glucose. Upon measuring RAW264.7 cell proliferation and NO production, PAP1-A manifested a potent immunomodulatory effect (12.5–100 μg/mL) with negligible cytotoxicity and was regarded as a promising ingredient in food supplementation [144]. The effect of (*Adiantum capillus-veneris* Linn.) leaf powder was investigated in a fish feeding study in which it was added to the diet and exhibited a pronounced effect on the immune system [145].

### 4.8. Antimicrobial and Antiviral Effects

In the quest for new antimicrobial agents to combat antimicrobial resistance, ferns have been largely unexplored. The skin lysosomal activity and superoxide dismutase activity were raised compared to the control in the groups receiving the dietary fern *Adiantum capillus-veneris* Linn. Additionally, a skin bactericidal effect was reported against *Escherichia coli* (EHEC ATCC 43895), Klebsiella, *Aeromonas hydrophila*, *S. aureus*, *Pseudomonas aeruginosa*, and *Escherichia coli* (CI) [145]. The sugar compositions in the nectaries produced by 101 fern species from the family Polypodiaceae were appraised as a valuable food source since it attracted scale insects, ants, and snails and other opportunistic animals [146] (Figure 10) (Table S1). One study delt with the essential oils from six *Pyrrosia* species and their in vitro antimicrobial activity with 2,4-pentadienal, phytol, and nonanal as the major components detected by GC-MS. From this, the *Pyrrosia lingua* extract was reported to have the highest potency against *S. aureus* (ATCC 25923) at 2.5 μL/mL [147].

### 4.9. Nutritional Importance of Ferns

New sources of human diet have never been more in demand, and edible ferns in regions such as East Asia and North America have been in use for a long period of time. Compared to leafy vegetables such as spinach and rockets, the carotenoids content estimated in ferns belonging to 13 families was about 1.2 to 4 times the former and equivalent to 4.77 mg⋅g^−1^ DW, with particularly high levels of lutein and *β*-carotene. These latter pigments are of vital importance in retinal tissue. In Dryopteridaceae species, *β*-carotene contributed the highest percentage to the total carotenoid content, up to 14 times that in cabbage. *β*-Carotene is a powerful antioxidant and provitamin A that serves to protect the skin [148].

In China, fern use as a traditional food date back to more than 3000 years, with *Pteridium aquilinum* var. latiusculum and *Osmunda japonica* recorded as the most widely eaten ferns by Chinese people together with *Athyrium multidentatum* (Doll.) [119]. Many parts of these edible ferns are used, for instance, the fronds, stems, rhizomes, and young leaves, where their starch content could be extracted, processed, and used for cooking. Fern cakes are popular in China from this starch, and many plant parts are stir-fried with chicken or meat [146].

In the Russian Far East diet, the ostrich fern (*Matteuccia struthiopteris* (L.) Tod.) and bracken fern (*Pteridium aquilinum* (L.) Kuhn) were the most popular with a valuable long-chain fatty acid content up to 5.5 and 0.5 mg/g dry weight for ARA and EPA, respectively. Ferns are known for their long-chain polyunsaturated fatty acid content such as arachidonic acid, eicosapentaenoic acid, sciadonic acid, juniperonic acid, and others; thus, they were postulated as a possible ingredient in formulating vegetarian diets [149]. *Athyrium multidentatum* (Doll.), *Pteridium aquilinum* (L.) Kuhn, and *Osmunda cinnamomea* Linn were assessed as widely ingested potherbs in China to show that their nutritive values are rich in protein, carbohydrates, minerals, and fats; additionally, they possess effective antioxidant effects and biomolecule-protective actions based on their total phenolic and flavonoid contents [119]. Just like many mushrooms and plants, *Diplazium maximum* (D. Don) was used as a food by Western Himalayas tribes [11]. The dried young fronds exhibit a high dietary fiber content, essential amino acids, up to 50% PUFAs, crude proteins, and phenolics. Further studies on its applicability as a food product revealed the favorable dispersibility and swelling properties, which prepare DM to be a part of instant food preparations [120].

The nutritive content of European ferns belonging to eight families, namely, Aspleniaceae, Athyriaceae, Dennstaedtiaceae, Dryopteridaceae, Onocleaceae, Osmundaceae, Polypodiaceae, and Thelypteridaceae, was investigated with respect to fatty acids, phenolics, ascorbates, and carotenoids and revealed that these plants’ young fronds were comparable in their nourishment profile to commonly used vegetables as spinach and others sold in the market with a desirable n-6/n-3 ratio between 2 and 6.4. The antioxidant effects of most of these fiddleheads in the ORAC assay were above 1 g Trolox equivalent per gram/dry weight [149].

The hazardous consequences of the use of some ferns were elaborated despite their use as a cultural edible herb. Among which, *Diplazium esculentum* (Koenig ex Retz.) consumed by the Rajbanshi population after cooking displayed both immunotoxic and hemolytic activities in a dose-dependent manner [150]. The *Matteuccia struthiopteris* (L.) Tod. plant was the cause of acute poisoning and GIT symptoms upon its raw ingestion despite its documented health benefits, yet little beyond being destroyed by boiling was known about the toxic component [150].

Beyond the risk of ptaquiloside inclusion in ferns, most of the toxicities seem minor and reversible, although we recommend further in-depth studies to confirm this observation. *Polypodium leucomotos* was marketed as Fernblock^®^ with no genotoxicity, oral toxicity, or mutagenicity effects. Regarding the genus Dryopteris, several acylphloroglucinols from its rhizomes were reported as anthelminthics and caused blindness in cattle, yet rat in vivo studies revealed no death associated with these extracts up to a dose of 5000 mg·kg^−1^. Even the potential nephron- and hepatoxicities caused at subchronic doses were reversible [151]. Similarly, *Dryopteris crassirhizoma* Nakai caused no genotoxicities up to 2000 mg·kg^−1^ in bacterial chromosomal aberration and bone marrow tests. *Macrothelypteris torresiana* (Gaudich) Ching was safe under the same dose and showed no hematological or biochemical changes. It is worth mentioning that appropriate cooking and frequent non-daily consumption could lower any toxicity risk as along as toxic ptaquilosides and analogs are absent [152].

## 5. Fern Use in Cosmetics

Many fern extracts demonstrated promising effects as cosmetic agents, for example, the volatile components of *Matteuccia struthiopteris* roots, trophophyll, and petioles [153]. *Ophioglossum vulgatum* L. was characterized by its rich flavonoids and polyols and formulated as a facial cleanser in the form of pickering emulsion stabilized by carbon dots (CDs-Arg) [154]. Ag nanoparticles (AgNPs) of the vegetable fern (named Gosari) revealed strong in vitro antioxidant potential [155]. Ferns such as bird’s nest extracts are composed of a substantial amount of *p*-coumaric acid and fucose-rich mucilage, which contribute to their antiaging effect via oxidative stress reduction. Several reports documented the role of polysaccharides in retaining the moisture and elasticity of skin [156]. The genus Asplenium produced a volatile profile with a predominant shikimic, terpene, and lipoidal nature, especially *Asplenium onopteris* in which 4-hydroxybenzoic acid represented 8.7% with a potential antiaging action [157,158]. *Microsorum grossum* (Polypodiaceae) extract rich in phytoecdysteroids such as 20-hydroxyecdysone was tested for its skin-protective effect using a human fibroblast model, gene expression and modulation study, and SIPS test, which indicated its efficacy and regulation of heme oxygenase 1 (HO1) to reduce oxidative stress that contributes to skin aging [159]. *Polypodium leucotomos* extract was suggested to be included in daily cosmetic preparations due to its antioxidant effect based on its caffeic acid and ferulic acid, which cured ultraviolet-induced erythema. Furthermore, the extract improved cell membrane integrity and elastin expression. Its future applications might be in photoprotection or its combined administration with sunscreens [160].

## 6. Application of Modern Extraction Methods to Ferns

Extraction methods and their efficiency are the foundation of good natural products research. Several novel techniques have started to gain scientific attention during the last few years, and their impact might significantly advance fern plant extractions and yield such as microwave-assisted extraction and supercritical fluid extraction. Dryopteris fragrans was extracted for its volatile oils using 1-Ethyl-3-methylimidazolium acetate as a digesting agent and microwave extraction at 79 degrees C for 3.6 min with a plant mass of 0.9 g to produce the same yield reported before but in a faster and more solvent- and sample-efficient way [161]. Phloroglucinol extraction was enhanced from Dryopteris fragrans via microwave-assisted extraction combined with ionic liquid-based surfactant, and 1-octyl-3-methylimidazolium bromide presented the highest relative extraction efficiency at 50 °C, time 7 min, and irradiation power 600 W [162]. *Pteris multifida* essential oil was extracted using CO_2_ supercritical fluid extraction and steam distillation to provide a total of twenty-seven and forty-five components, respectively [163]. The *Cibotium barometz* phospholipid content was measured to check the quality of the extract. The content of phosphatidylcholine was 0.198% and was utilized as the benchmark level. The method involved applying supercritical fluid extraction with a temperature of 46.2 °C, pressure at 31.5 MPa, and a 0.37 L/kg modifier [164].

## 7. Ethnopharmacological Uses of Ferns

Ferns have been widely used ethnopharmacologically in different regions (Table 2). *Dryopteris crassirhizoma* was added to the pharmacopeial preparation Fufang Qingdai Wan in 40 g amounts based on its soothing and cooling effect to treat and resolve dermal macules. This formulation was prescribed for rashes, itching, erythema, psoriasis, and sores. Moreover, the rhizomes killed worms and helped cure abdominal pain caused by worm infection. The genus *Microsorum*, known in the Pacific Islands as “Metuapua’a”, was used in the treatment of several ailments, particularly renal diseases, as a diuretic and anti-inflammatory agent [165]. Since only a few pharmacological studies have been conducted so far on ferns, many of these bioactivities were not correlated to their chemical active ingredients except for a few such as exdysteroids, which are widely recognized as anti-inflammatory agents. Another example is the Chinese remedy called “Gusuibu” formed of a unique combination of *Pseudodrynaria coronans*, *Drynaria fortunei*, *Davallia divaricata*, *Davallia solida*, *Humata griffithiana*, and *Davallia mariesii* [165] that was given to collagen carriers to increase bone formation in skull defects with more than 90% new bone formation compared to the active control [166]. “Anapsos” was registered in Spain after clinical trials as “Regender” and “Armaya fuerte” containing the extracts of *Polypodium leucotomos* extracts to treat dermatitis and psoriasis [167]. Fernblock could play an important role as a nutraceutical agent as it was shown to act as an immunomodulator, photoprotectant, and antioxidant [168]. Similarly, Fernblock was prepared from the aqueous extract of *Polypodium leucotomos* [169].

## 8. Future Aspects and Long-Term Avenues

Ferns are among the most unexploited species on earth despite their richness in unusual chemical skeletons and bioactivities. The world is facing several challenging problems that require every effort to solve these global issues, which include but are not limited to the antibiotic resistance problem. The WHO announced that the discovery of natural products is a strategic approach to limit antibiotic resistance. Moreover, ferns are traditionally known for their antibacterial effect and represent a rich repository of drug leads.

This review highlighted the intense discoveries made so far in fern research during the previous six years and the outcomes. The focus on ferns’ rare molecules of chemical classes such as acylphloroglucinols, terpenes, and phenols with superior antimicrobial, antiviral, and antifungal activities paves the way for the further development of antibiotic resistance solutions. Currently, little literature data are available about ferns, which contradicts the actual chemical and biological potential of these vascular plants. Another perspective is the nutraceutical use of ferns in the diet. The various fern species available and those used by locals will be collected, extracted, and analyzed for their chemical components. Their antimicrobial as well as their cytotoxic effects will be measured to select safe and effective species that could contribute based on their in vivo and in vitro experimental results to a healthy diet for people, particularly those suffering from diabetes, cardiovascular ailments, and obesity.

## 9. Conclusions

In this review, more than 25 acylphloroglucinols were compiled from fern data, and their antidepressant activity was shown. More than 40 terpene compounds were described here, and the majority are reported to have an acceptable safety profile, with only the kaurene diterpenes in Pteris dispar demonstrating a moderate cytotoxicity between 6 and 10 µmol/L. Around 50 phenolics were documented with promising activity in the binding sites of many enzymes such as tyrosinases, *α*-amylase, *α*-glucosidase, and acetyl cholinesterases. The inherent antioxidant potential of polyphenolic compounds predisposes them to combat cellular oxidative stress, which forms the pathological principle of many diseases such as stress-induced liver damage, cardiovascular problems, atherosclerosis, diabesity, Alzheimer, and immune disorders.

Terpenes, followed by phenolics, exhibited the largest number of isolated active compounds. Regarding the neuroprotective effect, the *Psilotium*, *Polypodium*, and *Dryopteris* species possessed as their major phenolic components unique chemical moieties including catechins, procyanidins, and bioflavonoids. Likewise, sesquiterpenes from Pteris species such as pterosin B exhibited a neuroprotective effect 200 times that of ginsenoside Rg1, yet the mechanism of this activity was unrevealed until now.

While the highest antiproliferative activity was manifested by liglaurates at 0.17 μM, an antiadipogenic effect was seen for osmundolactone and angiopterosides with 30–20% inhibition rates of TG in 3T3-L1 cells. Unmatching was observed between traditional uses and experimental study outcomes; for instance, Polypodium vulgare was used to treat kidney disorders, yet it was reported as an antioxidant. *Dryopteris crassirhizoma* was used as an antibacterial agent and recently proved to be antiallergic.

## Figures and Tables

**Table 2:** The ethnopharmacological uses of ferns in different regions.

Ferns	Usage	Country/Region	Symptoms Treated	Reference
*Microsorum*	Traditional Pacific Island medicine	The Pacific	Treats various ailments, particularly renal diseases, as a diuretic and anti-inflammatory agent	[165]
*Dryopteris crassirhizoma*	Pharmacopeial preparation component (Fufang Qingdai Wan)	China	Treats dermal macules, rashes, itching, erythema, psoriasis, and sores	[165]
*Pseudodrynaria coronans*, *Drynaria fortunei*, *Davallia divaricata*, *Davallia solida*, *Humata griffithiana*, *Davallia mariesii*	Chinese formula (Gusuibu)	China	Promotes bone formation in skull defects	[165,166]
*Polypodium leucotomos*	Clinical trial drug (Anapsos)	Spain	Treats dermatitis and psoriasis	[167]
*Polypodium leucotomos*	Nutraceutical preparation (Fernblock)	Spain	Used as an immunomodulator, photoprotectant, and antioxidant	[168,169]

## Data Availability

No new data were created or analyzed in this study. Data sharing is not applicable to this article.

## Supplementary Materials

The following supporting information can be downloaded at: https://www.mdpi.com/article/10.3390/plants13182668/s1, Table S1. Biological activities and potencies of compounds and/or extracts of different fern species.
