# Supplementary material for: Insights into Chemical Diversity and Potential Health-Promoting Effects of Ferns
Source: Plants (Basel). 2024 Sep 23;13(18):2668. doi: 10.3390/plants13182668 (PMC11434777; doi:10.3390/plants13182668)
Supplement: Supplementary file 1 [file plants-13-02668-s001.zip › plants-3141792-supplementary.pdf]

Table S1. Biological activities and potency of compounds and/or extracts of different fern species.

No.	Effect	compound /extract	Bioactive components	Species	Potency/Experimental model	References
1	antinflammatory	aerial part extract	/	*Athyrium multidentatum*	1.in-vitro lipopolysaccharide (LPS)-induced inflammatory model<br>2.in-vivo lung tissue	[118]
2	antinflammatory and antioxidant	fronds ext.	polyphenols	*Matteuccia struthiopteris, Osmundajaponica, Matteuccia orientalis and Pteridium aquilinum*	1. inhibition of (IL1-β) roots of O. japonica (IC50 of 17.8 μg/mL) young fronds of M orientalis (50.0 μg/mL)<br>2. interleukin-6 (IL6), and the gene expression of iNOS by LPS-elicited macrophage 50% decrease in M. orientalis ext.<br>3. DPPH<br>4. ABTS	[117]
3	antioxidant	fronds extract	total polyphenols of 51.69 mg/g, flavonoids of 58.05 mg/g in mature sterile fronds	*Stenochlaena palustris*	1. ferric reducing power.<br>2.radical scavenging activity 72.51	[119]
4	antioxidant	fronds extract	total flavonoid content is about 14.33%.	*Dryopteris erythrosora*	1. acetylcholinesterase effect more than 90%<br>2. ABTS (0.36 mg/ml)<br>3.DPPH (similar to rutin)<br>4.superoxide anion scavenging<br>5.FRAP (comparable to rutin)	[120]
5	antioxidant	ethanol extracts	total phenolic (476.52 ± 11.26 mg GAE per g extract) and total flavonoid (924.81 ± 4.25 mg RNE per g extract	*Osmunda cinnamomea Linn, Pteridium aquilinum and Athyrium multidentatum*	1. (DPPH)15.01 μgmL−1<br>2. ABTS (EC50 of 4.63 μgmL−1)<br>3. OH radical ((EC50 of 48.01 μg mL−1)<br>4. FRAP (EC50 of 13.20 μgmL−1)	[121]
6	high protein, nutritive and PUFA up to 50%	young fronds hydroalcoholic ext	high contents of dietary fibre (38.32 g/100 g dry basis) and crude protein (25.39 g/100 g dry basis)	*Diplazium maximum*	1. IC50 values in DPPH (48.49 ± 0.94 lg/mL)<br>2. ABTS (22.16 ± 0.25 lg/mL) assays	[122]
7	Antioxidant	extracts rich in phenolics	phenolics reach up to 16.9 mg GAE/g	*Asparagus officinalis* L. *Spears*	1. CUPRAC, μM TE 27.58 2. FRAP, μM TE, 13.33	[124]

8	Antioxidant	subterreanean parts aerialparts	Flavonoid Content (TFC)24.63± 1.34% Total Phenolic Content (TPC) 9.58±0.41%,	*Stenoloma chusanum*	1. DPPH 12.5-100 µg/mL<br>2. 98% of the ABTS radicals were inhibited<br>3. Tyrosinase inhibitory activity 137.48±2.67µg/mL.	[125]
9	antioxidant	extracts rich in carotenoids	average total phenolic content 163 mg GAeqv·g−1 DW	*Asplenium, Athyrium, Blechnum, Davallia, Pteridium, Dryopteris, Polystichum, Marsilea, Regnellidium, Matteuccia, Osmunda, Polypodium, Adiantum, Lastrea, Phegopteris, Thelypteris, and Cystopteris*	Most of the extracts showed<br>1. ORAC 0.5 g Trolox eq/gm dry weigh<br>2. DPPH assays IC50 is less than 30 µg·mL-1	[126]
10	antioxidant	extracts rich in flavonoids	high total flavonoid content 10.74 ± 0.25 mg/	*Isoetes sinensis Palmer*	1. (DPPH) 4.8 mg/µL<br>2. (ABTS) 3.2 mg/µL<br>3. (Superoxide radical) 42.6 mg/µL	[127]
11	antioxidant	water exts	total flavonoid extract (180 mg/kg, i. g.)	*Pteris multifida*	1. positive anti-BPH effect<br>2.decreased VEGF and bFGF expression but enhanced TGF-b1 expression,<br>3. reduce the levels of serum testosterone and dihydrotestosterone and increase estradiol/testosterone ratio.	[86]
12	antiinflammatory	ethanol extracts	phloroglucinol derivatives and triterpenes	*Dryopteris crassirhizoma* (DC)	1. suppression of Th2 cytokine overproduction<br>2. suppression of mast cell infiltration and reduction of nasal fluid Treg cytokines	[129]

13	antioxidant	fronds methanolic ext	high polyphenolic content	*Polypodium vulgare* L.	1.cellular repair activity in 3T3 fibroblast cells<br>2. phototoxicity, ROS production and cytoprotective effects against oxidative stress<br>3. negligable cytotoxicity at physiological concentrations	[130]
14	antiinflammatory	hydroalcoholic ext	flavonoids, alkaloids, tannins, saponins, and terpenoids	*Adiantum capillus-veneris linn*	1. inhibition of NF-κB-P65 synthesis<br>2.ACVL plant extract + CBZ protects the MDA level from the elevation.<br>3. prevents the reduction in both GSH content and SOD activity<br>4. 3β-HSD and 17β-HSD in ACVL plant extract + CBZ-treated rats recorded a normal level	[131]
15	antioxidant	water fractions	high 3-Deoxyanthocyanidins,	*Adiantum, Dryopteris, Blechnum, Pteris , Azolla species*		[132]
16	antioxidant	70%ethanolic ext	flavonoids-enriched extracts	*Pteris ensiformis Burm.*	1.higher in vitro scavenging activity against superoxide anion radical and hydroxyl radical than crude extracts.<br>2.higher antiproliferative activity of flavonoids-enriched extracts against MCF-7 and HepG-2 cell lines.	[133]

17	antiadipogenic	polar fractions	phenolic compounds	*Adiantum capillus-veneris*	1. in-vitro efficiency against α-amylase/α-glucosidase.<br>2. inhibited the pancreatic lipase enzyme (PL). 0.8µg/mL<br>3. inhibited PL in vitro with an ascending order of PL-IC50 values (lg/mL): ferulic acid; 0.48± 0.06< ellagic acid; 13.53 ± 1.83<chlorogenic acid; 38.4± 2.8<A. capillus-veneris; 1600±100	[137]
18	antiadipogenic	methanolic and aqueous ext	rich procyanidin-type A content	*Tectaria coadunata*	inhibits α-glucosidases and B-amylase	[47]
19	antiadipogenic	polar fractions	total phenolics (TP) and hydroxycinnamic acids (THC)	*Stenochlaena palustris*	inhibitor of α-glucosidase and amylase enzymes EC50 2.9 lg/mL	[138]
20	antitumor	60% ethanolic ext.	1. total phenolic 476.52mg GAE per g extract<br>2. flavonoids were and 924.81mg RNE per g extract	*Osmunda cinnamomea* Linn. *Pteridium aquilinum* and *Athyrium multidentatum* (Doll.).	1. (MMP) reduction in HepG2 cells 2. morphological changes 3. Apoptosis<br>4. 60% decrease in cellular proliferation	[121]
21	neuroprotective	ethanolic extracts	high phenolic and flavonoid content	*Diplazium esculentum* (Retz.)	reduced the BACE-1 and amyloid beta 42 (Aβ42) peptides	[141]
22	neuroprotective	methanolic and aqueous ext	rich procyanidin-type A content	*Tectaria coadunata*	anticholinesterases	[47]
23	skin health	polar fractions	aromatic polyphenols, phenolic acids content in BNEE	*Asplenium australasicum* (J. Sm.) *Hook*	1. supressed tyrosinase and pigmentation<br>2. reduce UV-induced erythema	[144]
24	antitumor	18% and 27% depressing ratio at 50 and 100 mg/ml,	fucose-rich polysaccharide mucilage rich fractions	*Asplenium australasicum (*J. Sm.*) Hook*	antiproliferative	[144]
25	skin health	methanolic frond ext	polyphenols	*Polypodium leucotomos fern*	photoprotective for skin, hair and nail reduction in UV-induced erythema	[145]

26	skin health	alcoholic ext and aqueous	procyanidin typeA	T. *coadunata*	tyrosinase inhibitory activity 149.41 mg kojic acid equivalent (KAE)/g	[47]
27	immuno modulat ory	water exts	PAP1-A polysacchari de	*Pteridium aquilinum*	RAW264.7 cells proliferation and NO production,	[146]
28	immuno modulat ory	fronds powder	phenolics and terpenoids, alkaloids, quaternary and N-oxides, fiber and elements (e.g. Zn and Cu) indicating	*Adiantum capillus-veneris*	enhanced immune system and fish growth rate	[147]
29	antimicr obial	fronds powder	phenolics and terpenoids, alkaloids, quaternary and N-oxides, fiber and elements (e.g. Zn and Cu) indicating	*Adiantum capillus-veneris*	1. skin lysosomal activity and superoxide dismutase<br>2. skin bactericial	[147]
30	antimicr obial	essential oils	2,4-pentadienal, phytol and nonanal	P. *tonkinensis,* P. *lingua,* P. *davidii,* P. *gralla,* P. *porosa,* P. *subfurfuracea*	P. lingua revealed MIC of 2.5 µL/mL against Staphylococcus aureus (ATCC 25923)	[149]
